# Nanotuner targeting mitochondrial redox and iron homeostasis imbalance for the treatment of acute liver injury

**DOI:** 10.7150/thno.119357

**Published:** 2025-08-16

**Authors:** Minghao Li, Qiwei Yang, Jie Gao, Xudong Liu, Jihua Shi, Wenzhi Guo, Yi Zhang, Qiwen Yu, Xinzhi Sun, Shuijun Zhang

**Affiliations:** 1 Department of Hepatobiliary and Pancreatic Surgery, Henan Key Laboratory of Digestive Organ Transplantation, The First Affiliated Hospital of Zhengzhou University, Zhengzhou, China; 2 Zhengzhou Key Laboratory for Hepatobiliary & Pancreatic Diseases and Organ Transplantation, Zhengzhou, China; 3 Department of Orthopedics, The First Affiliated Hospital of Zhengzhou University, Zhengzhou, China; 4 Department of Surgery, The First Affiliated Hospital of Zhengzhou University, Zhengzhou, China.

**Keywords:** acute liver injury, nanotuner, mitochondrial targeting, ferroptosis, labile iron pool

## Abstract

**Background**: Acute liver injury (ALI), a highly perilous clinical condition triggered by diverse etiological factors, frequently progresses to acute liver failure with life-threatening consequences. However, due to the limited intervention time window for ALF, donor shortages, challenges in utilizing marginal grafts, surgical complexity and risks, and the high economic burden, developing novel therapeutic strategies targeting ALI-induced ALF has become imperative.

**Methods**: Through transcriptome analysis, we determined that ferroptosis is a key driver in the pathogenesis of ALI. To combat hepatocyte ferroptosis, we designed a novel mitochondria-targeted nanotuner (CPTD) for regulating mitochondrial oxidative stress and iron homeostasis imbalance during ALI. This nanotuner features a cerium oxide (CeO₂) nanozyme core with a polydopamine (PDA) coating, functionalized with triphenylphosphonium (TPP) for mitochondrial targeting and deferoxamine (DFO) for iron chelation. In vitro and in vivo experiments evaluated CPTD's ability to target mitochondria and the labile iron pool (LIP).

**Results**: The nanotuner demonstrates dual regulatory capacity by effectively accumulating in hepatic mitochondria to concurrently scavenge reactive oxygen species (ROS) and sequester labile iron ions, thereby rectifying mitochondrial oxidative stress and iron dyshomeostasis. Comprehensive evaluations across multiple ALI models, mainly including hepatic ischemia-reperfusion injury and acetaminophen-induced hepatotoxicity, revealed that CPTD robustly inhibits ferroptosis, mitigates oxidative damage, attenuates inflammatory responses, and preserves hepatic function.

**Conclusions**: Our findings establish this dual-targeting nanotuner as a promising therapeutic strategy for ALI, providing novel insights into mitochondrial redox and iron homeostasis modulation.

## Introduction

Acute liver injury (ALI), a critical clinical syndrome characterized by sudden hepatic dysfunction, which often progresses to acute liver failure (ALF) without timely intervention, poses a serious threat to human health 1,2. The etiologies of ALI are diverse, encompassing drug toxicity, ischemia-reperfusion injury (IRI), viral/bacterial infections, and metabolic disorders, among others 3. Liver transplantation remains the most appropriate treatment for severe cases 3,4. However, due to the limited intervention time window for ALF, donor shortages, challenges in utilizing marginal grafts, surgical complexity and risks, and the high economic burden, developing novel therapeutic strategies targeting ALI-induced ALF has become imperative.

Ferroptosis is a newly discovered form of cell death characterized by iron-dependent lipid peroxidation leading to mitochondrial and cellular collapse, is associated with a variety of ALI, such as hepatic ischemia reperfusion injury (HIRI) and drug-induced liver injury (DILI) 5-8. When excessive Fe²⁺ accumulates intracellularly, it forms the labile iron pool (LIP), a redox-active dynamic iron reservoir 9. Fe²⁺ within the LIP acts as a catalyst for the Fenton reaction. It is a critical driver of oxidative stress and ferroptosis, where intracellular ferrous iron (Fe²⁺) reacts with hydrogen peroxide (H₂O₂) to generate highly reactive hydroxyl radicals (·OH) 10. The Fe²⁺/Fe³⁺ redox cycling in the Fenton reaction amplifies cytotoxic reactive oxygen species (ROS) production and exacerbates oxidative stress, overwhelming antioxidant defense systems such as glutathione peroxidase 4 (GPX4) 11. The ensuing ROS storm aggravates mitochondrial dysfunction and hepatocyte ferroptosis. The regulation of ferroptosis by iron metabolism hinges critically on the control of the LIP capacity 12. Mitochondria serve as central regulators of iron homeostasis, where iron primarily supports oxidative phosphorylation (OXPHOS) 13. The accumulation of the mitochondrial LIP (mLIP) further disrupts redox balance, directly leading to impaired OXPHOS, thereby exacerbating cellular dysfunction. In HIRI, reperfusion disrupts iron homeostasis by releasing excess mLIP. This iron overload enhances mitochondrial ROS (mROS) production, leading to mitochondrial lipid peroxidation (mLPO) and membrane rupture 14. Research has shown that inhibition of ferroptosis can effectively reduce the degree of HIRI in marginal grafts. In aged livers, iron ion accumulation was more pronounced 15. APAP hepatotoxicity involves NAPQI-mediated depletion of glutathione and subsequent inhibition of iron oxidation through GPX4. Targeting 7-dehydrocholesterol reductase (DHCR7), a regulator of ferroptosis, could effectively prevent APAP-induced ALF in mice 16. These findings establish LIP homeostasis as a pivotal nexus connecting iron dysregulation, oxidative stress, and ferroptotic hypersensitivity. Targeting mitochondria oxidative stress and iron dyshomeostasis provides broad prospects for clinical translation in the treatment of ALI patients.

Recently, metal nanoenzymes with bioenzymatic mimetic activity have shown promising applications in the treatment of diseases. For example, manganese nanoenzymes can effectively alleviate ALI, platinum nanoenzymes are used in the treatment of ischemic stroke, and metal-organic frameworks (MOF) promote healing of diabetic ulcers 17-19. Among these nanozymes, Cerium-based nanozymes have garnered significant attention due to their excellent biocompatibility and antioxidant properties, enabled by the redox cycling of Ce³⁺ (reduced) and Ce⁴⁺ (oxidized), and have been found to effectively treat diseases such as stroke, acute kidney injury, and rheumatoid arthritis 20-22. Cerium oxide (CeO₂) nanozymes effectively scavenges superoxide anions (·O₂⁻), hydrogen peroxide (H₂O₂), and hydroxyl radicals (·OH), offering distinct advantages over traditional protein-based enzymes, including lower cost, higher stability, and ease of modification 23. Although CeO2 nanozymes have been shown to be effective in scavenging a variety of ROS, it is difficult for CeO_2_ to regulate iron ion effectively. Therefore, it is expected that CeO_2_ can be further modified to enhance its ability to regulate iron metabolism, so as to achieve a dual regulatory effect on oxidative stress and iron homeostasis imbalance during ferroptosis.

**Scheme 1:**
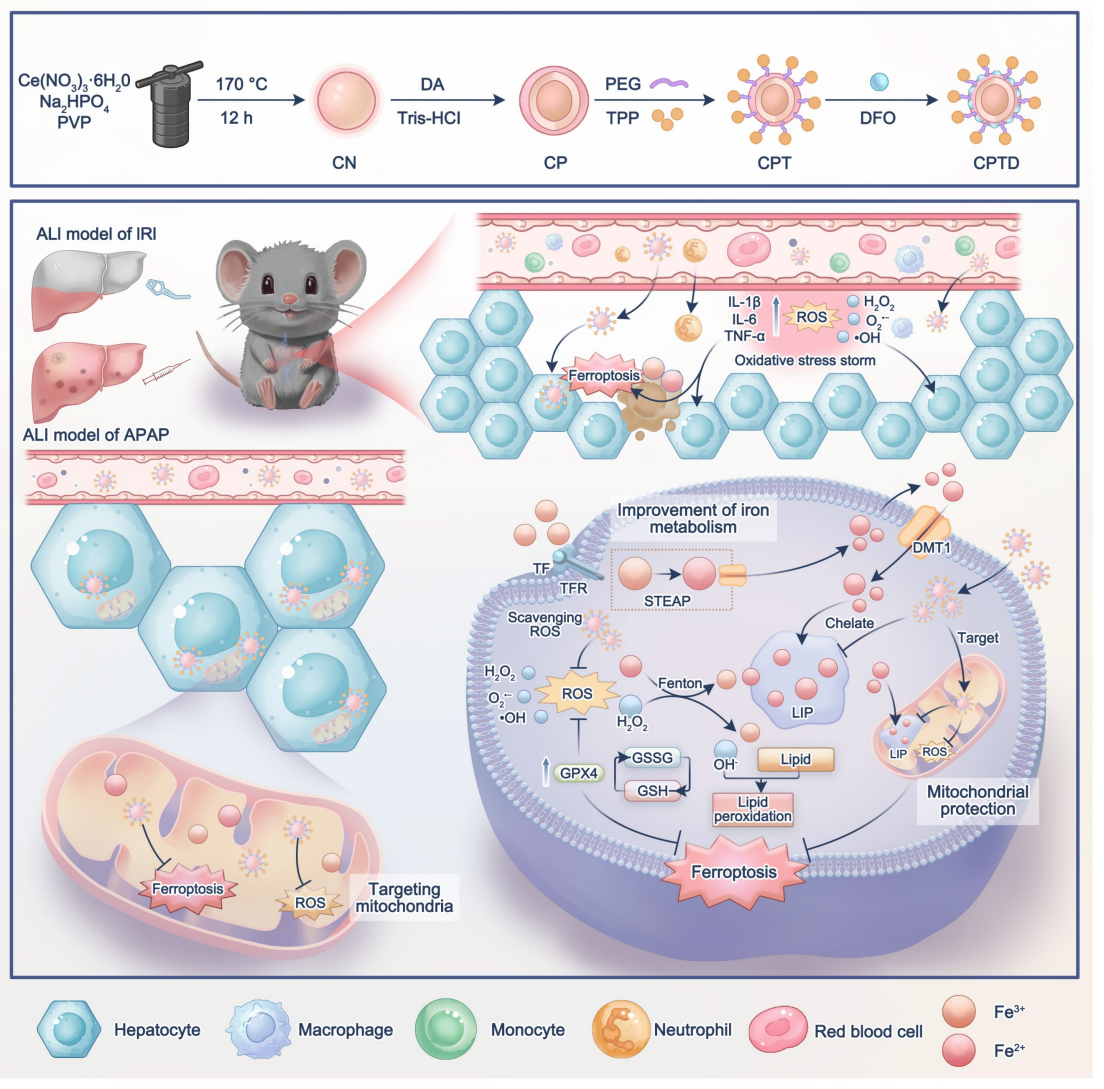
Schematic illustration of mitochondria-targeted Nanotuner for treating ALI by combating oxidative stress and sequestering labile iron ions. After entering hepatocytes, CPTD preferentially aggregates around mitochondria, inhibiting oxidative cascades and ferroptosis signaling activation through scavenging mROS and sequestering mLIP, thereby maintaining intracellular redox homeostasis and correcting iron metabolism imbalance during ALI. CN: CeO_2_ nanoparticle; CP: CN coated with polydopamine; CPT: CP modified by TPP; CPTD: CPT loaded with DFO.

In this study, we first demonstrated the significance of ferroptosis in HIRI and APAP-induced ALI. We then constructed a dual nanotuner (CPTD) targeting ferroptosis (Scheme 1). The core of CPTD is composed of CeO₂, and its surface is coated with polydopamine (PDA). Owing to the abundance of phenolic hydroxyl groups, amino groups, and carbonyl groups, PDA enhances the ability of CeO₂ to scavenge ROS, endows it with iron chelation ability, and facilitates subsequent surface functionalization. Next, the mitochondrial targeting group triphenylphosphonium (TPP) and the iron ion chelator deferoxamine (DFO) were modified on the surface. When CPTD is targeted to the mitochondria of ferroptosis hepatocytes, the mitochondrial oxidative stress environment degrades PDA to achieve the responsive release of DFO. Subsequently, the exposed CeO₂ core and released DFO exert dual scavenging effects on mitochondrial ROS and iron ions. By regulating mitochondrial oxidative stress and iron homeostasis imbalance, CPTD protects hepatocytes from ferroptosis. We confirmed that CPTD was able to reduce liver injury, alleviate oxidative stress, and inhibit ferroptosis in both HIRI and APAP-induced liver injury mouse models. Our study suggests that our constructed nanotuner targeting mitochondrial oxidative stress and iron homeostasis imbalance may represent a universal therapeutic strategy for ALI.

## Results

### Active ferroptosis signaling in ALI

**Figure 1:**
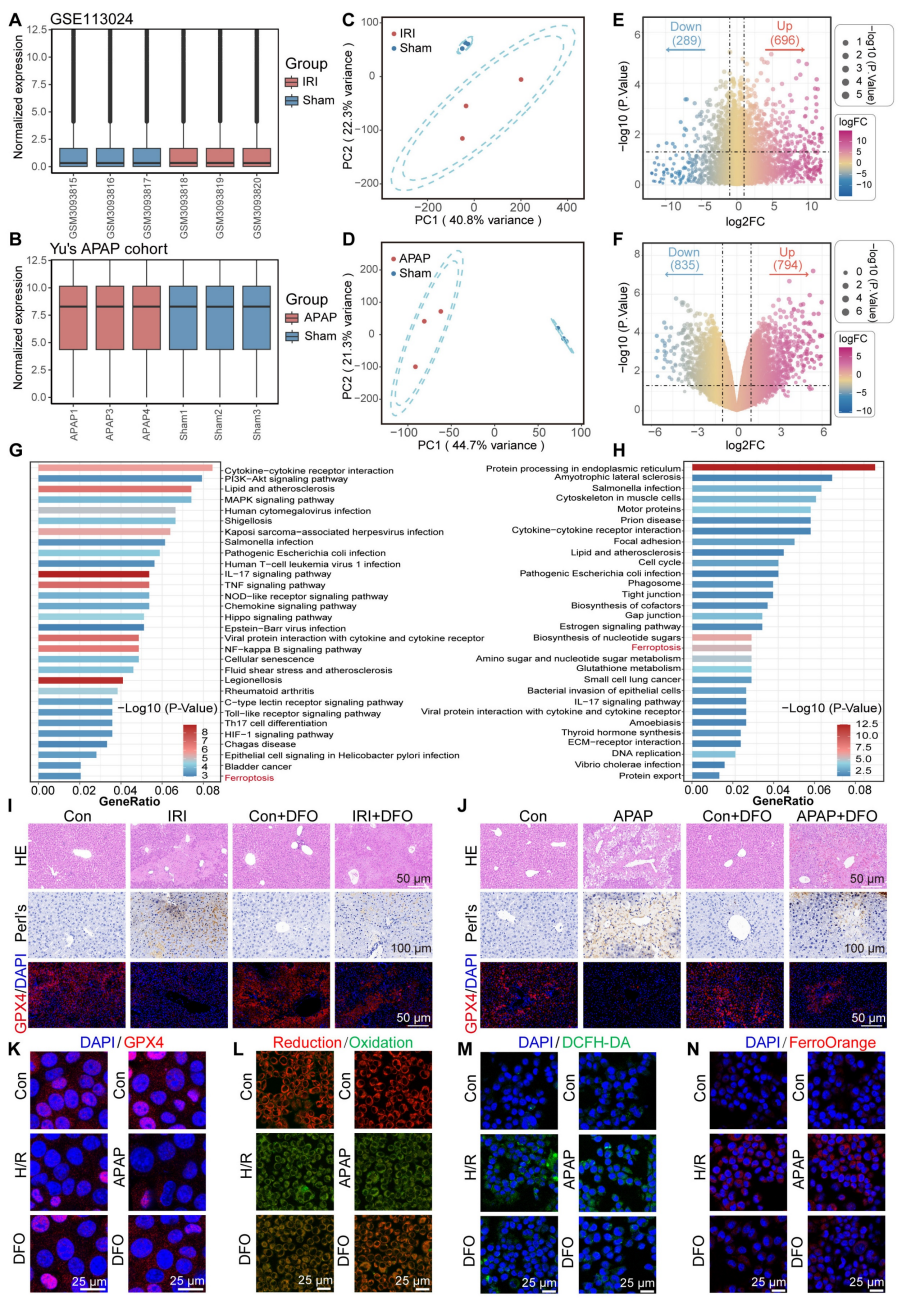
Bulk transcriptome analysis revealed active ferroptosis signaling in ALI. (**A-C**) Normalized dataset of hepatic IRI (A), PCA plot (B), and volcano plot of DEGs (C). (**D-F**) Normalized dataset of APAP induced ALI (D), PCA plot (E), and volcano plot of DEGs (F). (**G**) The KEGG enrichment analysis bar plot of the IRI cohort. (**H**) The KEGG enrichment analysis bar plot of the APAP induced ALI cohort. (**I**) HE staining, Prussian blue staining, and IF analysis of GPX4 in liver tissue of DFO-pretreated mice after hepatic IRI. (**J**) HE staining, Prussian blue staining, and IF analysis of GPX4 in liver tissue of DFO-pretreated mice after APAP induced ALI. (**K-N**) Cellular levels of GPX4 (**K**), LPO (**L**), ROS (**M**), and Fe^2+^ (**N**) after stimulation of AML12 cells by H/R or APAP in the presence or absence of DFO pretreatment.

To elucidate the critical contribution of ferroptosis pathway activation in acute liver injury, here, we selected two representative models of ALI, including hepatic ischemia-reperfusion injury (GSE113024) and acute APAP-induced ALI 24. First, the independent cohort data are normalized to make the sample characteristics comparable (Figure 1A-B). Next, principal component analysis (PCA) indicated high intergroup variability and excellent intragroup consistency in each independent cohort, which suggested that the data were reproducible (Figure 1C-D). Subsequently, between-group difference analysis was performed in each of the two datasets. With *P* < 0.05 as the screening criterion, 696 up-regulated genes and 289 down-regulated genes were identified in the GSE113024 cohort. A total of 794 up-regulated genes and 835 down-regulated genes were found in Yu's cohort (Figure 1E-F). Kyoto En-cyclopedia of Genes and Genomes (KEGG) enrichment analysis demonstrated potential pathways involved in ALI by differentially expressed genes (DEGs). We visualized the top 30 pathways with significant contributions in ALI. In HIRI, DEGs were found to be mainly enriched in PI3K-AKT, MAPK, NF-kappa B, and HIF-1 signaling pathways. And significant upregulation of Lipid and atherosclerosis, Glutathione metabolism pathway was found in APAP-induced ALI. Interestingly, significant activation of the ferroptosis pathway was found in two different ALI events (Figure 1G-H). These results implied that oxidative stress and ferroptosis are common mechanisms in ALI. Therefore, inhibiting oxidative stress and targeting ferroptosis may be a prospective therapeutic strategy for ALI.

Subsequently, we verified whether there was an increased ferroptosis driven by iron overload during ALI by using mouse models. In the ALI models, hematoxylin-eosin (HE) staining revealed significant hepatocellular necrosis with nuclear consolidation in the liver. Prussian blue staining suggested a large amount of iron deposition in the necrotic areas. Immunofluorescence displayed that GPX4 expression was significantly down-regulated in the ALI models (Figure 1I-J). Of note, the iron chelator DFO partially reversed cell death in ALI, as evidenced by a reduction in necrotic area, attenuated iron deposition, and restoration of GPX4 expression. It suggested that a disturbance of iron metabolism and increased ferroptosis events occurred during ALI (Figure 1I-J). Furthermore, we used DFO to validate the changes that occurred in cells during ALI. Confocal laser scanning microscope (CLSM) observed a significant down-regulation of GPX4 expression and an increase in lipid peroxidation (LPO) after treatment of AML12 cells with hypoxia/reoxygenation (H/R) or APAP (Figure 1K-L; Figure S1A-D, Supporting Information). Obviously, DFO restored GPX4 expression and reduced LPO accumulation. These results provided further evidence that ferroptosis is an important mechanism of cellular injury during ALI.

Ferroptosis is caused due to accumulation of lethally toxic membrane lipid peroxides induced by overloading of LIP and is an important mechanism of cellular injury in ALI 25,26. When AML12 cells were struck by H/R or APAP, significant reactive oxygen species accumulation and Fe^2+^ deposition occurred (Figure 1M-N; Figure S1E-H, Supporting Information). Fe^2+^ overload aggravates oxidative stress by promoting ROS generation and intracellular ROS accumulation on the one hand, and aggravates membrane lipid peroxidation by participating in the Fenton reaction to generate excess free radicals on the other hand, leading to cellular damage 27. Interestingly, pretreatment of AML12 cells with DFO prior to their injury significantly reduced intracellular ROS and Fe^2+^ levels. This was attributed to the excellent iron chelating ability of DFO, which led to a reduction in intracellular free iron and thereby modulation of LIP. Inspired by this, the dual-action approach of controlling free iron accumulation and scavenging ROS holds promise as a therapeutic strategy for ALI.

### Construction and characterization of CPTD

**Figure 2:**
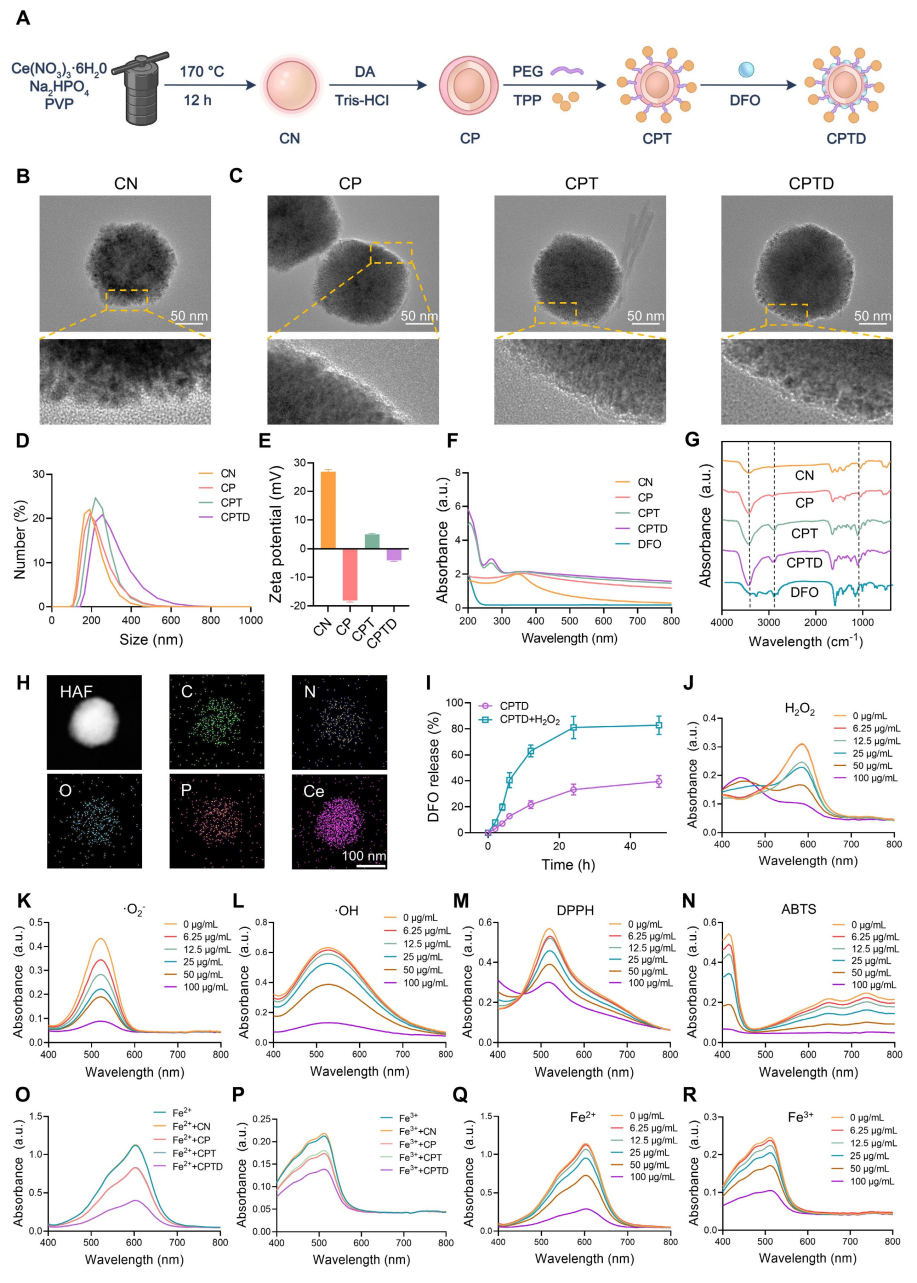
Synthesis and characterization of CPTD. (**A**) Preparation process of CPTD. (**B-C**) HRTEM images of CN, CP, CPT and CPTD. (**D-E**) Hydrodynamic particle sizes (**D**) and zeta potential (**E**) of CN, CP, CPT and CPTD (n = 3). (**F-G**) UV-Vis absorption (**F**) and FTIR spectra (**G**) of CN, CP, CPT, CPTD and DFO. (**H**) STEM image of CPTD and element mapping of C, N, O, P and Ce. (**I**) Release kinetics of DFO from CPTD with or without H_2_O_2_-simulated oxidative stress. (**J**) UV-Vis absorption spectra of H2O2 degradation with different concentrations of CPTD. (**K**) The SOD-like capacity of CPTD evaluated by UV-Vis absorption spectroscopy of ·O_2_^-^ scavenging. (**L**) UV-Vis absorption spectra of the ·OH scavenging capacity of CPTD as determined by the salicylic acid-ferrous sulfate method. (**M-N**) Antioxidant capacity of CPTD as determined by the DPPH (**M**) and ABTS (**N**) methods. (**O-P**) UV-Vis absorption spectra of intracellular Fe^2+^ (**O**) and intracellular iron (**P**) chelating capacity of different materials. (**Q-R**) UV-Vis absorption spectra of intracellular Fe^2+^ (**Q**)and intracellular iron (**R**) chelating capacity at different concentrations of CPTD.

Firstly, we synthesized CeO_2_ nanoparticle (CN) and performed a series of modifications on CN to construct CPTD with mitochondrial targeting, reactive oxygen species scavenging, and iron ion chelation functions. Specifically, we synthesized CN using hydrothermal method and further coated its surface with PDA to form CP. Next, PEG and TPP were modified on the surface of CP to form CPT, which endowed the nanoparticles with a targeting function towards mitochondria. Finally, DFO was loaded on the CPT surface to construct CPTD (Figure 2A). We documented the solution color changes during stepwise synthesis: hydrothermally synthesized CN appeared milky white, while PDA-coated CP, CPT, and CPTD solutions turned slate-gray (Figure S2A-B). High-resolution transmission electron microscopy (HRTEM) revealed that the synthesized CN consisted of uniformly sized, well-dispersed polygonal nanoparticles (Figure 2B). A distinct 5~10 nm PDA coating layer was observable on CP surfac. Furthermore, no significant morphological alterations occurred following DFO loading (Figure 2C). Dynamic light scattering (DLS) was used to measure the hydrated particle size and zeta potential of the nanoparticles. The particle sizes of CN, CP, CPT, and CPTD were 204.1 ± 8.5 nm, 217.3 ± 8.0 nm, 244.06 ± 4.4 nm, and 257.7 ± 6.9 nm, respectively (Figure 2D; Figure S3A, Supporting Information). Then we measured the particle size and polymer dispersity index (PDI) of CPTD in different solvents continuously for 3 days using DLS to verify its stability. The particle size and PDI of CPTD did not change significantly in H_2_O, PBS, and DMEM + 10% FBS, which indicated that CPTD exhibited excellent stability in different solvents (Figure S4A-B, Supporting Information). The zeta potential of CN was 26.9 ± 0.7 mV, and after surface coating with PDA, the zeta potential decreased to -18.1 ± 0.5 mV, which was due to the negative charge carried on the surface of PDA. After further modification of TPP, the zeta potential of CPT increased to 4.9 ± 0.4 mV, which to some extent indicates the successful modification of TPP. After loading with DFO, the zeta potential of CPTD was -3.9 ± 0.4 mV (Figure 2E). We used X-ray diffraction (XRD) to evaluate the crystal structure of CN, CP, CPT and CPTD, and the results showed that the diffraction peaks of CN were consistent with the standard card (PDF#43-1002), indicating the successful synthesis of CN (Figure S5A, Supporting Information). CP, CPT, and CPTD also exhibited similar diffraction peaks, indicating that the crystal structure of the core was retained (Figure S5A, Supporting Information).

X-ray photoelectron spectroscopy (XPS) was employed to evaluate the elemental composition, cerium valence states, and bonding environments across different samples. The results revealed that synthesized CN contained C, N, O, and Ce elements, while CP exhibited peak profiles similar to CN (Figure S6A, Supporting Information). Notably, no significant morphological alterations occurred following PDA coating. Systematic XPS analysis provided critical evidence for structural stability: consistent primary peaks in CP and CN spectra confirmed core integrity; Ce3d spectra showed negligible changes post-PDA coating; and the absence of significant peak splitting in CPT/CPTD Ce3d spectra. The attributable to increased "shell" thickness limiting XPS probing dept, which further validated progressive functionalization (Figure S6B, Supporting Information). Subsequent peak deconvolution of Ce 3d spectra investigated cerium valence distribution, revealing Ce³⁺/Ce⁴⁺ ratios of approximately 0.943 in CN and 1.058 in CP, demonstrating preservation of cerium valence distribution essential for CPTD's antioxidant functionality (Figure S6C-D, Supporting Information). The stable formation of the CP core-shell architecture is paramount for successful CPTD functionalization. PDA's abundant primary/secondary amine functionalities substantially enhance its inherent adhesiveness, providing fundamental support for uniform coating on CP surfaces. Concurrently, π-π stacking within the PDA network crosslinks with PVP chains, reinforcing coating stability. XPS analysis confirmed metal-phenolic coordination between PDA and Ce: CN exhibited Ce³⁺ characteristic peak areas of 18.65% at 882 eV and 15.37% at 901 eV, while CP demonstrated increased areas of 21.25% (882 eV) and 19.11% (901 eV), which representing 13.9% and 24.3% expansions, respectively (Figure S6C-D, Supporting Information). This binding energy-specific peak enlargement directly originates from phenol coordination bond formation. The elevated Ce³⁺ content further indicates coordination-stabilized reduced cerium states. Bonding alterations in elemental spectra confirmed PDA incorporation: C1s displayed increased C-O ratios; N1s revealed increased N-H peaks; O1s showed expanded O=C and O-H regions (Figure S6E-J, Supporting Information). Thermogravimetric analysis (TGA) showed that the mass loss of CP was 4.7% higher than that of CN throughout the heating process, which was attributed to the decomposition of PDA. Notably, CPTD ultimately showed a mass loss 23.3% higher than that of CPT (Figure S7A).

To further confirm the successful modification of the components, ultraviolet-visible spectroscopy (UV-Vis) and Fourier transform infrared spectrometer (FTIR) were used to characterize each nanoparticle. CPT showed two different absorption peaks at 220-300 nm compared to CP, representing successful PEG and TPP modification, respectively, while CPTD showed DFO-specific amide absorption at 220-240 nm, indicating successful loading of DFO (Figure 2F). CP showed increased phenolic hydroxyl group stretching vibration in the range of 3200-2700 cm^-1^ (Figure 2G), and coating of PDA produced a broad absorption band compared to CN indicating successful modification of PDA (Figure 2F). CPT showed increased C-O and C-H vibration in the range of 1000-1400 cm^-1^ indicating successful modification of PEG and enhanced C-H stretching vibration of benzene ring in the range of 3100-2800 cm^-1^ indicating successful modification of TPP. CPTD showed enhanced N-H stretching vibration in the range of 3500-3300 cm^-1^ range exhibited enhanced N-H stretching vibration indicating successful loading of DFO (Figure 2G). We calculated the encapsulation efficiency and loading efficiency of DFO to be 56.8 ± 3.9% and 22.1% ± 0.1%, respectively, by measuring the absorbance of the DFO/Fe^3+^ complex at 420 nm (Figure S8A-D, Supporting Information). The calculated DFO loading efficiency aligns with the additional mass loss observed for CPTD compared to CPT in thermogravimetric analysis (TGA) results. TEM elemental mapping shows that our final synthesized CPTD retains the C, N, O, and Ce elements from CN and the P element from TPP (Figure 2H). In conclusion these results indicated that we successfully synthesized CN and further modified PDA, PEG, TPP, and loaded DFO on its surface. Due to PDA can be degraded in the presence of H_2_O_2_, we further investigated the drug release properties of CPTD in the presence of H_2_O_2_. We found that the release rate of DFO was significantly accelerated in the presence of H_2_O_2_, implying that CPTD was able to release DFO responsively in an oxidative stress environment (Figure 2I).

### The capacity of CPTD for scavenging diverse free radicals and chelating iron ions

After verifying the successful synthesis of CPTD, we further investigated its free radical scavenging and iron ion chelating properties. First, we evaluated the H_2_O_2_ degradation ability of CPTD. By detecting the characteristic absorption spectra of the complexes of Fe^3+^ and xylenol orange at 540-570 nm it was found that CPTD efficiently degraded H_2_O_2_ in a dose-dependent manner (Figure 2J). Similarly, the characteristic absorption peaks of ·O_2_^-^ and ·OH decreased with increasing CPTD incubation concentration, suggesting that CPTD can mimic a variety of antioxidant enzymes such as catalase (CAT) and superoxide dismutase (SOD), with spectral antioxidant capacity (Figure 2K-L). Besides, we further evaluated the antioxidant potential of CPTD by DPPH and ABTS methods. UV-vis absorption spectroscopy revealed that the characteristic absorption peaks of DPPH and ABTS decreased in a concentration-dependent manner with increasing CPTD concentrations, indicating effective scavenging of free radicals (Figure 2M-N). On one hand, this is derived from the abundant reducing functional groups such as catechol and imine present in PDA; on the other hand, DFO itself possesses certain free radical scavenging capabilities, which can be attributed to the hydroxamic acid moiety in its structure, enabling the capture of free radicals 28. To better understand the contribution of each component, we evaluated the individual and combined radical-scavenging capabilities of standalone CN nanoparticles, free DFO, and their physical mixture. The results demonstrated that DFO exhibited moderate DPPH and ABTS radical scavenging activities, though significantly weaker than CN. DFO contributed negligibly to ·O₂⁻ elimination and showed minimal ·OH scavenging capacity (Figure S9A-D, Supporting Information).

The iron ions chelating ability of CPTD was evaluated to confirm the efficacy enhancement obtained after loading DFO. The Fe²⁺ chelating ability of CPTD was evaluated by measuring the characteristic absorption peak of the remaining Fe²⁺ complexed with tripyridyltriazine after chelation with different components. The Fe³⁺ content was determined by quantifying the characteristic absorption peak of the complex formed between phenanthroline and Fe²⁺, which was generated through the reduction of the remaining Fe³⁺. We found that the iron chelating performance of CPTD was obviously superior to that of CN, CP and CPT (Figure 2O-P). And CPTD chelated iron ions in a dose-dependent manner (Figure 2Q-R), indicating that the iron chelating performance of CPTD coated with PDA and loaded with DFO was significantly enhanced.

### Cellular uptake and mitochondrial targeting capacity of CPTD

**Figure 3:**
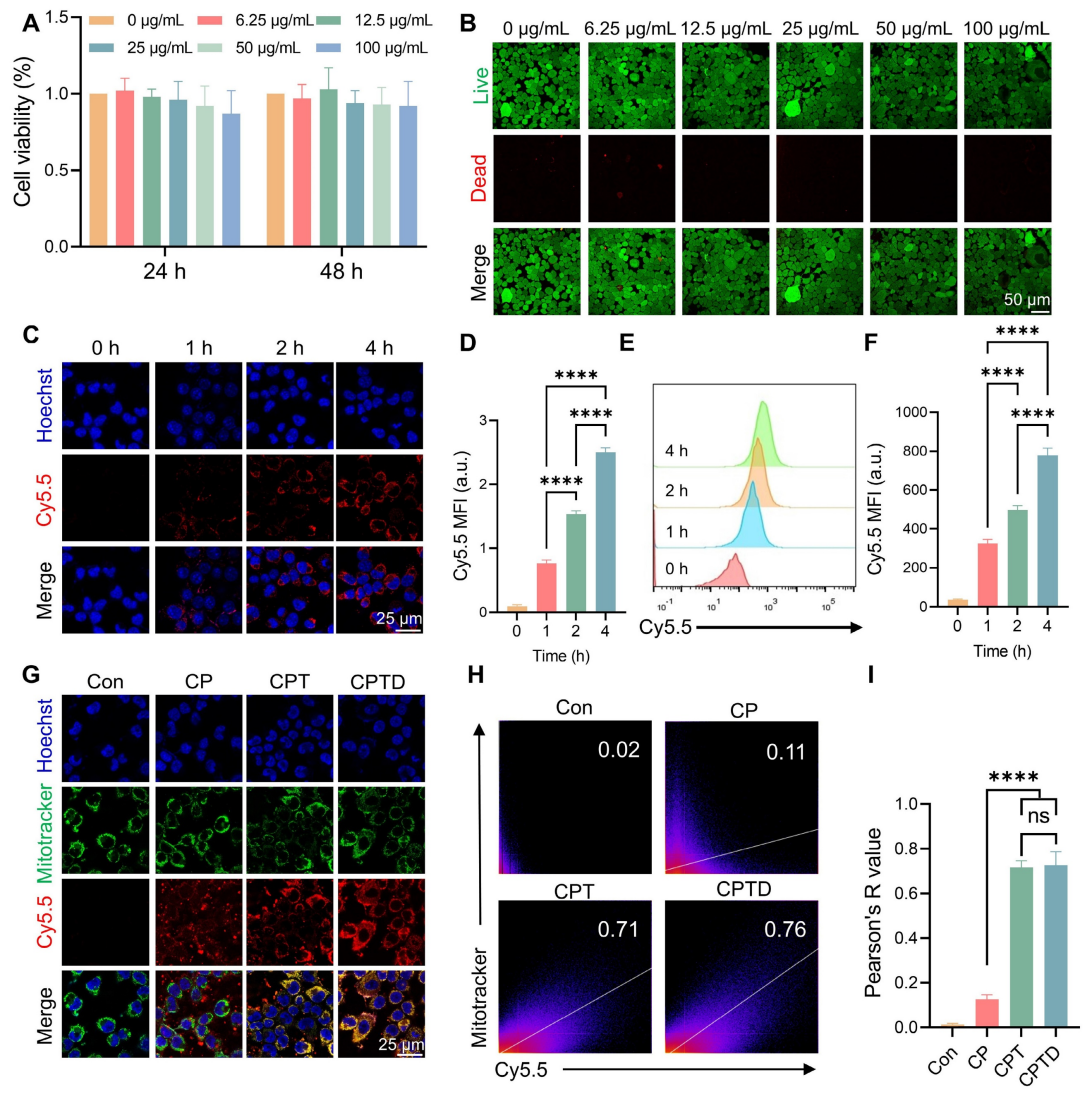
Cytotoxicity, cellular uptake and mitochondrial co-localization in vitro. (**A**) Cell viability of AML12 cells incubated with various concentrations of CPTD for 24 h and 48 h as determined by CCK-8 assay. (**B**) Live/dead staining of AML12 cells incubated with different concentration gradients of CPTD for 48 h. (**C-F**) Cellular uptake of CPTD labeled by Cy5.5 in AML12 cells at 0 h, 1 h, 2 h, 4 h detected by CLSM imaging (**C-D**) and flow cytometry (**E-F**) as well as quantitative analysis (n = 3). (**G**) Aggregation in mitochondria (Mitotracker (green)-labeled) of Cy5.5-labeled CP, CPT and CPTD in AML12 cells at 4 h detected by CLSM imaging. (**H**) Pearson's R value for Cy5.5 and Mitotracker fluorescence co-localization (**H**) and quantitative analysis (**I**, n = 3). Data are presented as mean ± SD, n.s. not significant, *p < 0.05, **p < 0.01, ***p < 0.001, and ****p < 0.0001 (one-way ANOVA with Tukey's post hoc test).

The toxic effects of CPTD on AML12 cells were investigated by assessing the viability of AML12 cells after treatment with different concentrations of CPTD. Cell Counting Kit-8 (CCK-8) assay showed no significant decrease in cell viability after treating AML12 cells at different concentration gradients for 24 or 48 h, indicating that CPTD has a favorable safety profile in vitro (Figure 3A). Similarly, Calcein AM (Calcein Acetoxymethyl Ester)/PI (Propidium Iodide) staining implied that CPTD did not significantly reduce the viability of AML12 cells (Figure 3B; Figure S10A, Supporting Information). Considering the potential delayed effects of material degradation products, we assessed the impact of long-term CPTD incubation on AML12 cell proliferation. Cell viability was evaluated using AM/PI staining after 1, 3, and 5 days of CPTD treatment. We observed no significant changes in proliferative activity across these time points (Figure S11A, Supporting Information). These findings demonstrate that CPTD induces neither significant cytotoxicity nor delayed toxicity at therapeutic doses. Furthermore, we investigated the efficiency of CPTD uptake by AML12 cells. Cy5.5-labeled CPTD can be observed as red fluorescence under CLSM after uptake by cells. It was co-cultured with AML12 cells and the fluorescence intensity was recorded at different times. We found that significant internalization of CPTD could be observed at 4 h of co-culture (Figure 3C-D). In addition, flow cytometry analysis revealed that fluorescence within AML12 cells increased with incubation time which indicated that CPTD was significantly internalized by AML12 cells (Figure 3E-F). The long-term intracellular distribution of nanoparticles is a topic of frequent discussion. To evaluate whether CPTD can be retained within cells, we conducted further experiments. After incubating AML12 cells with Cy5.5-CPTD, we observed a gradual increase in intracellular fluorescence over 24 h, demonstrating progressive accumulation of CPTD in AML12 cells (Figure S12A, Supporting Information). Interestingly, after 8 h, the fluorescence intensity plateaued, indicating cellular uptake saturation. This provides clear evidence that CPTD achieves prolonged intracellular retention in AML12 cells.

Then, to investigate whether CPTD has the expected mitochondrial targeting properties upon entering the cell, mitochondria were labeled with MitoTracker (green) to see whether CPTD co-localizes with AML12 cell mitochondria in the context of injury. CLSM observed that Cy5.5-labeled CPT and CPTD were predominantly distributed around mitochondria, which is direct visual evidence of CPT and CPTD targeting to mitochondria (Figure 3G). Successful modification of TPP enabled CPT and CPTD to have organelle tropism leading to their distinctive mitochondrial enrichment properties, which were not clearly observed in CP. The Pearson correlation coefficients of co-localization correlation analysis reached 0.73 ± 0.06 and 0.72 ± 0.03, respectively, which were significantly higher than 0.13 ± 0.02 in the CP group (Figure 3H-I). Mitochondria are the major site of cellular respiration, and the energy supply from OXPHOS enables cells to accomplish normal life activities 29. In addition, mitochondria are an important source of ROS, and oxidative stress damage and mitochondrial dysfunction are important causes of ALI cell damage 30. Therefore, CPTD can efficiently enter cells and enriched in mitochondria, which lays the foundation for the removal of mitochondrial ROS and iron and thus protects mitochondrial function.

### CPTD protects AML12 cells by disarming iron overload and maintaining redox homeostasis

**Figure 4:**
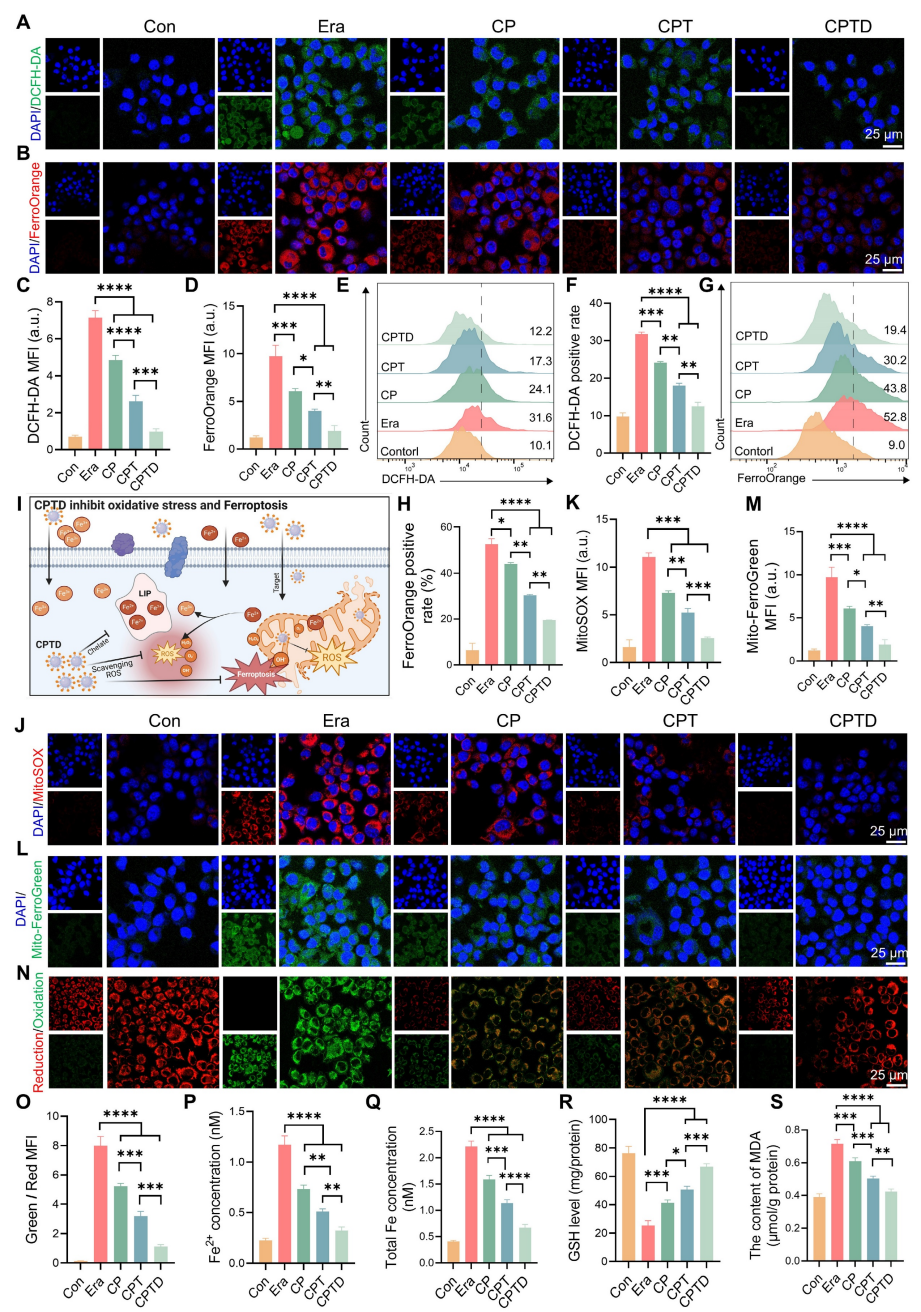
CPTD maintains redox homeostasis by counteracting oxidative stress damage and chelating free iron. (**A-B**) DCFH-DA staining for ROS levels (**A**) and FerroOrange staining for Fe^2+^ levels (**B**) of AML12 cells pretreated with or without erastin stimulation following treatment with CP, CPT or CPTD. (**C-D**) Quantitative analysis (n = 3) of DCFH-DA staining (**C**) and FerroOrange staining (**D**). (**E-F**) Flow cytometry (**E**) and quantitative analysis (**F**, n = 3) of DCFH-DA staining in each treatment group. (**G-H**) Flow cytometry (**G**) and quantitative analysis (**H**, n = 3) of FerroOrange staining in each treatment group. (**I**) Schematic illustration of CPTD scavenging ROS and stabilizing intracellular and mitochondrial LIP. (**J-K**) MitoSox staining for mROS levels (**J**) and quantitative analysis (**K**, n = 3) in each treatment group presented by CLSM. (**L-M**) MitoFerroGreen staining for intra-mitochondrial Fe^2+^ levels (**L**) and quantitative analysis (**M**, n = 3) in each treatment group presented by CLSM. (**N-O**) Cellular LPO levels (**N**) and quantitative analysis (**O**, n = 3) in each treatment group. (**P-Q**) Quantification of Fe^2+^ (**P**, n = 3) and total iron (**Q**, n = 3) levels in cells from each treatment group. (**R-S**) Quantification of GSH (**R**, n = 3) and MDA (**S**, n = 3) levels in cells from each treatment group. Data are presented as mean ± SD, n.s. not significant, *p < 0.05, **p < 0.01, ***p < 0.001, and ****p < 0.0001 (one-way ANOVA with Tukey's post hoc test).

Activation of ferroptosis is an important mechanism of cell injury in ALI. Intracellular iron is stored in the form of Fe^3+^ bound to ferritin, while Fe²⁺ forms the LIP 31. The accumulation of Fe²⁺ in the LIP during ALI triggers ferroptosis to accelerate cell fate termination 26. In addition, a significant portion of Fe^2+^ participates in the Fenton reaction leading to intracellular ROS accumulation. As a common inducer of ferroptosis, erastin acts through a mechanism related to the inhibition of the cystine/glutamate inverse transporter (System XC-) on cell membranes 32. Additionally, erastin can affect mitochondrial function through VDAC1 and alter mitochondrial membrane permeability, thereby interfering with iron metabolism and increasing oxidative stress 33. Here, the erastin-induced ferroptosis cell model was utilized to study the LIP regulation and ROS scavenging capacity of CPTD. We used 2 ', 7 ' - dichlorofluorescein diacetate (DCFH-DA) as an indicator of cellular ROS. Upon Erastin stimulation, the cells exhibited strong green fluorescence, indicating the accumulation of ferroptosis-associated reactive oxygen species (ROS). And this phenomenon could be effectively eliminated by CPTD (Figure 4A). Besides, CP and CPT also had a certain scavenging effect on ROS. FerroOrange fluorescent probes were employed to detect intracellular Fe^2+^ concentrations. Under erastin treatment conditions, the cells underwent a marked impairment of iron metabolism and a distinct red fluorescence was observed in CLSM. CP and CPT interventions resulted in a reduction of cellular oxidative stress, which in turn reduced F^2+^ accumulation in LIP. While CPTD reduced Fe^2+^ to almost normal levels (Figure 4B). Quantitative analysis revealed that ROS and Fe^2+^ levels decreased by 86% and 81%, respectively, after CPTD treatment (Figure 4C-D). Consistently, quantification by flow cytometry provides another piece of evidence. Better efficacy is harvested with CPTD treatment compared to CPT and CPT groups. During CPTD treatment, the area of green fluorescence detected by FITC was reduced, indicating significant removal of ROS (Figure 4E-F). The FerroOrange probe also captured significantly less positivity, confirming the excellent iron chelating ability of CPTD (Figure 4G-H). The structure of CPTD with cerium core, dopamine coating and DFO loading achieves a superposition of antioxidant properties and excellent iron chelation.

Extracellular free iron enters the cell either by binding to the transferrin receptor 1 (TFR1) or through the divalent metal ion transporter protein 1 (DMT1) 31. Fe^2+^ becomes part of the cytosolic or mitochondrial LIP, whereas Fe^3+^ is partially reduced to Fe^2+^, and the rest is bound to ferritin and stored. ROS outbreak is a key factor in ALI and contributes to the increase in lipid peroxidation, which ultimately leads to irreversible cellular damage. The mROS is the primary source of intracellular ROS, and mLIP is an important component of iron metabolism 13. During this period, the cell membrane or mitochondria are attacked by free radicals, leading to damage and dysfunction, imbalance of mitochondrial homeostasis, which triggers iron metabolism disorders, and excessive Fe^2+^ accumulates in the LIP 12,34. In addition, iron overload caused an imbalance in the antioxidant system, GPX4 activity was decreased, and the cells were unable to effectively defend against lipid peroxidation ultimately triggering ferroptosis (Figure 4I). Mitosox was applied to assess mROS levels under different treatment conditions, thus revealing enhanced mitochondrial targeting efficacy (Figure 4J-K). Mitochondria displayed a strong red fluorescent signal in erastin-induced injury. The decrease in mROS levels was incremental under CP and CPT treatments. While CPTD further decreased the mROS level. Furthermore, by using the Mito-ferroGreen probe, we observed an intense green fluorescence after exposure to erastin, which illustrated an increase in mitochondrial LIP under ferroptosis events. Likewise, the number of mLIP under CPTD treatment was less compared to CP and CPT treatment, indicating enhanced therapeutic efficacy of targeting mitochondrial (Figure 4L-M). The above results emphasize the synergistic therapeutic benefits of CPTD targeting mitochondria and chelated iron.

Immediately following this, we examined the degree of LPO in AML12 cells under different treatment conditions. The reduced and oxidized state products of the BODIPY probe were excited to red and green fluorescence, respectively, under CLSM to determine the extent of intracellular LPO. CLSM observed a significant increase in the degree of LPO under erastin induction, as evidenced by a clear predominance of green fluorescence. Notably, when receiving different treatments, the detected fluorescence presented a transition from green to orange and eventually to red predominance (Figure 4N). This implies that the LPO of CPTD-treated AML12 cells was significantly attenuated. CPTD significantly inhibited the increase of intracellular LPO with 2.48-fold inhibition efficiency of CP and 1.43-fold inhibition efficiency of CPT (Figure 4O). Additionally, we further examined intracellular Fe^2+^ and total iron concentrations. Intracellular Fe^2+^ and total iron concentrations in the erastin-induced ferroptosis scenario increased 4.09-fold and 4.41-fold, respectively, compared with the baseline levels in the control group, which reflected an obvious intracellular iron overload status. In contrast, the excellent iron chelating effect of CPTD significantly decreased the intracellular Fe^2+^ and total iron levels thereby inhibiting intracellular iron overload (Figure 4P-Q). Elevated intracellular GSH levels and reduced MDA levels also indicated that CPTD significantly inhibited LPO and maintained cellular redox homeostasis (Figure 4R-S). These findings demonstrated that the therapeutic strategy of targeting cellular and mitochondrial LIP could optimally attenuate erastin-induced cellular ferroptosis.

### CPTD regulates cell fate by targeting mitochondria and inhibiting ferroptosis

**Figure 5:**
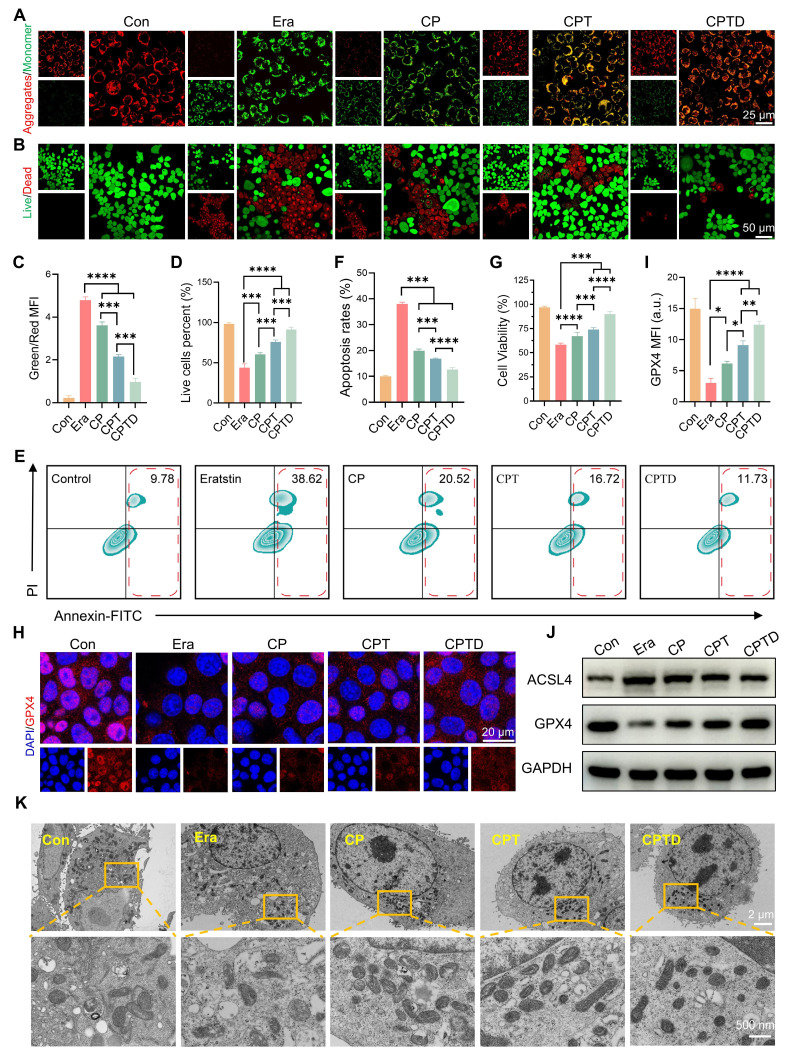
CPTD ameliorates mitochondrial dysfunction and protection of AML12 cells against ferroptosis. (**A**) JC-1 staining for MMP levels in each treatment group presented by CLSM. (**B**) Live/dead staining in each treatment group. (**C-D**) Quantitative analysis (n = 3) of the percentage for green/red fluorescence in JC-1 staining and the proportion of live cell pixels in live/dead staining. (**E-F**) Apoptosis in each treatment group as detected by flow cytometry (**E**) and quantitative analysis (**F**, n = 3). (**G**) Cell viability of AML12 cells in each treatment group as determined by CCK-8 assay. (**H-I**) IF analysis and quantification of GPX4 levels in each treatment group (n = 3). (**J**) Western blot analysis for protein expression levels of ACSL4 and GPX4 in cells of each treatment group (n = 3). (**K**) TEM images and localized magnifications of mitochondria in cells with or without erastin stimulation following treatment with CP, CPT, or CPTD. Data are presented as mean ± SD, n.s. not significant, *p < 0.05, **p < 0.01, ***p < 0.001, and ****p < 0.0001 (one-way ANOVA with Tukey's post hoc test).

Excessive production of superoxide radicals during ALI leads to mitochondrial dysfunction. Normal maintenance of oxidative phosphorylation depends on a polarized mitochondrial membrane. The mitochondrial membrane potential gradient serves as direct feedback for normal mitochondrial function. Hence, we detected the changes in mitochondrial membrane potential in different treatment groups by JC-1 probe. Under normal conditions, JC-1 aggregates in mitochondria to form multimers that produce intense red fluorescence, whereas when exposed to an injury situation, the decrease in mitochondrial membrane potential prevents the entry of JC-1 into the mitochondria and thus JC-1 monomers that are excited to intense green fluorescence are observed. After erastin-induced injury, the pronounced green fluorescence confirmed the disruption of mitochondrial integrity and the loss of the membrane potential gradient. The reduced mitochondrial membrane potential was restored to some extent by CP and CPT, and the effect of CPT was significantly superior to that of CP. Interestingly, the addition of CPTD led to a further restoration of mitochondrial membrane potential, which emphasized the therapeutic superiority derived from the enhanced mitochondrial targeting and ferroptosis inhibition effects (Figure 5A). Calcein AM /PI staining further reflected the protective effect of CPTD on injured AML12 cells (Figure 5B). Quantitative analysis showed that the ratio of green and red fluorescence produced by JC-1 decreased by 25.63%,55.11% and 79.75% in the CP, CPT and CPTD groups, respectively (Figure 5C). The CPTD-treated group also exhibited a more noticeable increase in the number of live cells compared to the other groups, which was almost equal to the control group (Figure 5D). Considering that the degree of apoptosis is an important indicator of the ability of CPTD to protect cells, we used Annexin V/PI co-staining assay to detect the reversal of erastin-induced growth inhibition of AML12 cells with different materials by flow cytometry. As shown in Figure 5E, erastin significantly enhanced apoptosis in AML12 cells, whereas CPTD treatment resulted in a significantly lower percentage of late apoptosis. Although the percentage of apoptosis was reduced to different degrees in all treatment groups, CPTD nevertheless displayed the most effective (12.59 ± 0.79) inhibition of erastin-induced apoptosis (Figure 5F). Furthermore, the CCK8 viability assay also demonstrated the most effective protection of AML12 cells when treated with CPTD (Figure 5G).

Next, since ferroptosis serves as a key mechanism of ALI cell injury, it is necessary to investigate the impact of CPTD on different core regulators of ferroptosis. As an important regulator of the antioxidant system, GPX4 is essential for maintaining cellular redox homeostasis 35. Upon erastin initiation of cellular ferroptosis progression, GPX4 expression levels decreased significantly, and a faint reddish glow of GPX4 staining can be observed by CLSM. This condition could be effectively rescued by CPTD, which we found to significantly rebound GPX4 expression (Figure 5H-I). To further verify the inhibitory effect of CPTD on ferroptosis, we examined the expression levels of several ferroptosis-related proteins via western blot (Figure 5J; Figure S13A, Supporting Information). Notably, erastin upregulated the expression level of the ferroptosis-enhancing factor acyl-coenzyme A (CoA) synthase 4 (ACSL4). CPTD supplementation effectively reduced the expression of ACSL4. Quite the contrary, CPTD administration was able to be a restoration of GPX4 expression. In addition, we observed the morphological changes of mitochondria in different conditions by TEM to reveal the direct mechanism of cell protection by CPTD (Figure 5K). In vitro exposure to erastin resulted in mitochondrial ultrastructural disruption in AML12 cells, as evidenced by mitochondrial shrinkage and thickening of mitochondrial membranes and ridges, which indicated the occurrence of ferroptosis. The CP-treated group still displayed marked vacuolization and cristae disruption. Mitochondrial morphology improved in the CPT-treated group. In the presence of CPTD, mitochondrial structures closely approximated those in the control group, with clearly visible cristae, which demonstrated CPTD's exceptional mitochondrial protective capacity.

It suggests that when AML12 is struck by ferroptosis, CPTD can efficiently aggregate around mitochondria to scavenge ROS and chelate iron. Altogether, the findings were exciting and demonstrated that CPTD targeting mitochondria and LIP provided significant benefits in protecting AML12 cells from oxidative stress injury and ferroptosis.

### CPTD is enriched in the liver and exhibits good biocompatibility

Given the promising antioxidant and ferroptosis-inhibiting efficacy of CPTD demonstrated in vitro, this motivates us to further explore its potential application in alleviating ALI in vivo. To investigate the distribution of CPTD in animals, we injected mice intravenously with Cy5.5-labeled CPTD and compared them with mice that received equal amounts of Cy5.5 treatment. To exclude the interference of other factors, the blank control was injected with an equal amount of saline. In vivo fluorescence imaging was performed on different groups of mice at 3h, 6h, 12h and 24 h after injection. We found that CPTD exhibited significant accumulation in the liver region compared to Cy5.5 injection alone at various time points, suggesting that CPTD can be effectively enriched in the liver (Figure S14A, Supporting Information). Subsequently, these mice were euthanized and major organs were removed for ex vivo fluorescence imaging. Evidently, Cy5.5-CPTD displayed a more persistent residence time and fluorescence intensity in the liver compared to free Cy5.5, followed by the kidney, indicating that CPTD can be effectively targeted to the liver (Figure S14B-C, Supporting Information). This was attributed to the characteristic distribution pattern of nanoparticles of this size 36.

Furthermore, the normal histological images of HE staining of the major organs in each group demonstrated that the CPTD had good biocompatibility (Figure S15A, Supporting Information). This was further evidenced by serologic indices, with negligible changes in ALT and AST (liver injury indicators), creatinine and BUN (kidney injury indicators), LDH and CK (heart injury indicators) induced by CPTD injections (Figure S15B-G, Supporting Information). The small size of metal nanoparticles facilitates their penetration through biological membranes, raising concerns about dose-dependent and cumulative toxicity. Achieving a balance between therapeutic efficacy and safety constitutes a major challenge for the clinical translation of nanomedicines. CeO₂ nanoparticles are gaining increasing prominence due to their favorable biocompatibility, and this expanding applicability underscores ceria's substantial clinical translation potential. Long-term biocompatibility assessments further bolster confidence in CPTD's clinical prospects. Our study demonstrates that intravenous administration of CPTD at therapeutic doses daily for three consecutive days induced no significant toxic effects. Histopathological examination of H&E-stained sections from the heart, liver, spleen, lungs, and kidneys at multiple post-injection time points (days 1, 3, 5, 7, and 14) revealed no significant inflammation, hemorrhage, necrosis, or other abnormalities (Figure S16A, Supporting Information). Additionally, serum levels of ALT, AST, Cr, BUN, LDH, and CK measured at the study endpoint (day 14) remained within normal ranges, confirming preserved renal, hepatic, and cardiac function (Figure S16B-G, Supporting Information).

### In vivo hepatic protection by CPTD in IRI-induced ALI

**Figure 6:**
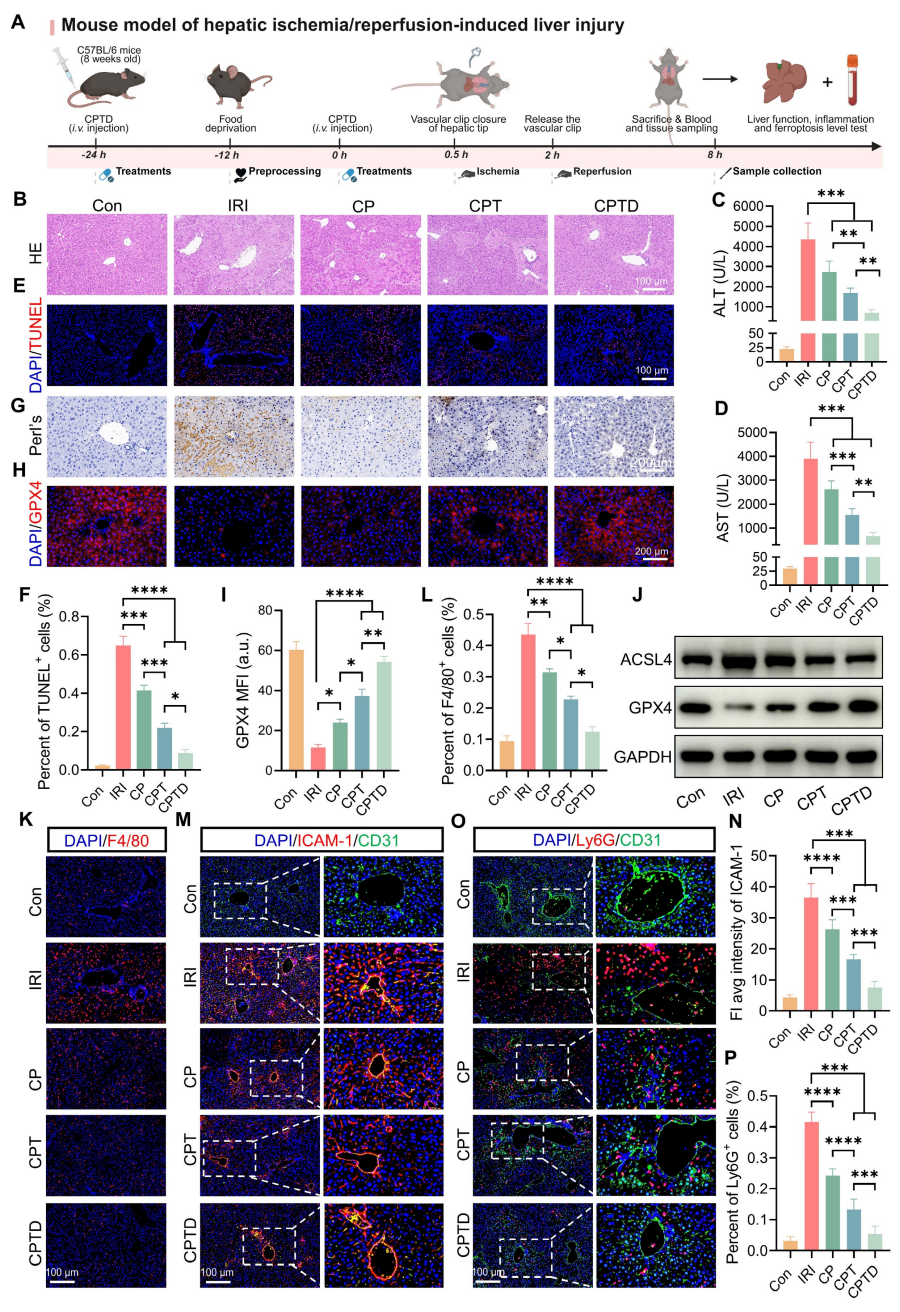
Therapeutic efficacy of CPTD in mouse model of IRI-induced ALI. (**A**) Schematic illustration for studying CPTD-mediated hepatocyte protection in the IRI mouse model. (**B**) Representative images of H&E staining of the liver. Liver injury (white dotted line) manifests as necrosis, nuclear consolidation, and cellular vacuolization. (**C-D**) Quantification of ALT and AST in serum of mice receiving or not receiving IRI following treatment with CP, CPT or CPTD (n = 6). (**E-F**) Representative images of TUNEL staining of liver sections (**E**) and quantitative statistics of the number of TUNEL-positive cells (**F**, n = 5). (**G**) Representative images of Prussian blue staining in liver sections (n = 5). (**H-I**) GPX4 fluorescence images of mouse liver sections (**H**) and quantification analysis (**I**) after treatment with various preparations (n = 5). (**J**) Western blot of ACSL4 and GPX4 in liver tissues of mice treated with different preparations (n = 3). (**K-L**) Representative images of F4/80 fluorescence in liver sections (**K**) and percentage of F4/80 positive cells (**L**) of mice in each treatment group (n = 5). (**M-N**) Representative images of ICAM-1/CD31 IF in liver sections (**M**) and quantification analysis of ICAM-1 fluorescence (**N**) of mice in each treatment group (n = 5). (**O-P**) Representative images of Ly6G/CD31 fluorescence in liver sections (**O**) and percentage of Ly6G positive cells (**P**) of mice in each treatment group (n = 5). Data are presented as mean ± SD, n.s. not significant, *p < 0.05, **p < 0.01, ***p < 0.001, and ****p < 0.0001 (one-way ANOVA with Tukey's post hoc test).

HIRI is the major reason for early graft dysfunction after liver transplantation and hepatic failure after liver resection 37. To further confirm the hepatic protection effect of CPTD on HIRI, we simulated HIRI by using the mouse model of hepatic tip blockade. Two doses of CPTD were administered intravenously in advance, at 24 h and 30 min before the occlusion period, followed by a 90-min blocking period to induce severe hepatic ischemia, and then 6 h of reperfusion (Figure 6A). As revealed by H&E staining, the liver tissue of IRI-induced ALI exhibited extensive hepatocellular necrosis (white dotted line), severe vacuolization, and inflammatory cell infiltration. In contrast, injection of CPTD reduced the necrotic area, protecting hepatocytes that would otherwise progress to necrosis and maintaining them in a state of edema (Figure 6B). The significant elevation of serum biochemical indexes ALT and AST reflected the presence of severe liver injury in the IRI model group, while CPTD injection led to a significant decrease in ALT and AST, achieving effective protection of liver function (Figure 6C-D). In addition, we observed a significant reduction in the number of TUNEL-positive cells in the livers of mice after CPTD treatment, from 64.8% in the IRI group to 8.7% in the CPTD-treated group (Figure 6E-F). These data provided evidence that CPTD was effective in blocking hepatocyte damage during ALI throughout the treatment period.

DHE staining visually demonstrated hepatic oxidative stress levels under different treatment conditions (Figure S17A, Supporting Information). Encouragingly, compared to the IRI group, CPTD treatment suppressed oxidative stress injury in hepatocytes during the damage process, as evidenced by a significant reduction in tissue superoxide accumulation (DHE fluorescence intensity was reduced by 83.8%) (Figure S17B, Supporting Information) and a marked decrease in lipid peroxidation levels (serum MDA levels was reduced by 52.8%) (Figure S18A, Supporting Information). Furthermore, CPTD effectively enhanced the levels of the antioxidants GSH and SOD in mice, which are essential for maintaining redox homeostasis (Figure S18B-C, Supporting Information). CPTD successfully restored the reduced GSH level during IRI to almost the same level as the control group (increased from 9.99 ± 1.56 μg/mg protein to 19.97 ± 1.02 μg/mg protein); SOD also increased from 31.00 ± 2.23 μg/mg protein in the IRI group to 52.17 ± 2.17 μg/mg protein in the CPTD group. Notably, supplementation with CP and CPT also ameliorated HIRI damage to varying extents, but their therapeutic efficacy was significantly inferior to that of CPTD.

Next, the inhibitory effect of CPTD on ferroptosis was further validated in the mouse model. We assessed the distribution of Fe^3+^ in the region of IRI by Prussian blue staining. We observed that IRI mice accumulated large amounts of Fe^3+^ in the injured liver region (Figure 6G). This phenomenon arises mainly from two mechanisms: on one hand, sinusoidal endothelial cell damage during reperfusion leads to leakage of circulating iron into the hepatic parenchyma; on the other hand, disordered iron metabolism caused by hepatocellular homeostasis imbalance results in increased iron retention 31,38. After CPTD treatment Fe^3+^ accumulation was significantly reduced, which could be associated with the iron chelating properties of the targeted LIP and the ability to inhibit LPO overactivation. Furthermore, immunofluorescence staining revealed that CPTD effectively enhanced GPX4 expression in liver parenchyma (Figure 6H-I). Mice subjected to IRI exhibited a significant increase in ACSL4 expression and a marked decrease in GPX4 expression. Compared with other component-treated groups, CPTD treatment demonstrated more effective modulation of iron metabolism-related pathways, as evidenced by a notable upregulation of GPX4 expression and a reduction in ACSL4 expression (Figure 6J; Figure S19A, Supporting Information). In summary, CPTD exhibited excellent iron suppression. The burst of ROS and iron retention are key drivers of ferroptosis and LPO. Our meticulously designed CPTD integrates three functions, targeting mitochondria, antioxidation and iron chelation which enables a two-pronged therapeutic strategy to ameliorate oxidative stress and stabilize intracellular iron pools. This approach prevents ferroptosis during IRI by restoring the antioxidant system and reducing LPO.

When HIRI leads to the initiation of ALI, circulating monocytes/macrophages are immediately recruited, and Kupffer cells resident in the hepatic microenvironment are activated and release large amounts of inflammatory mediators, which undoubtedly aggravate hepatocyte injury 37,39. We found that the proportion of monocytes/macrophages aggregated in the liver with F4/80 as a representative marker was greatly reduced after CPTD treatment (Figure 6K-L). Meanwhile, the expression levels of inflammatory mediators IL-1β and IL-6 decreased by 57.8% and 57.9%, respectively (Figure S20A-B, Supporting Information). Pretreatment with CPTD impeded massive macrophage recruitment during IRI, and the downregulation of inflammatory mediators also suggests that CPTD plays an important role in inhibiting suppression of macrophage activation. The alterations in liver nonparenchymal cells compel us to keep exploring whether CPTD could remodel the hepatic inflammatory microenvironment. Hepatocellular injury during IRI is typically accompanied by inflammatory cell infiltration. Neutrophil adhesion and recruitment are among the early events occurring during hepatic IRI, which help assess the extent of tissue damage 40,37. Intercellular cell adhesion molecule-1 (ICAM-1), as an endothelial inflammatory marker, plays a crucial role in mediating neutrophil-endothelial cell interactions and neutrophil migration 41. By labeling vascular endothelium with CD31, ICAM-1 and Ly6G staining can effectively determine vascular endothelial injury during IRI and the inflammatory response induced by transcellular migration. Pretreatment with CP and CPT partially reduced ICAM-1 expression, as well as perivascular endothelial neutrophil recruitment (Figure 6M-N). Notably, the same dose of CPTD was more effective in ameliorating endothelial injury, and the number of Ly6G-positive neutrophils was further reduced, confirming a deeper reduction in neutrophil adhesion and infiltration (Figure 6O-P). Following I/R injury, expression levels of NLRP3 and phosphorylated NF-κB (p NF-κB) were significantly increased. Notably, compared to the model group, CPTD treatment reduced NLRP3 levels and suppressed I/R-induced NF-κB phosphorylation (Figure S21A-C, Supporting Information). Specifically, CPTD effectively inhibited the upregulation of inflammatory signaling post-I/R injury, which aligns with the observed reduction in inflammatory cell recruitment. These findings indicated that the capacity of CPTD to inhibit inflammatory cell activation would reduce the inflammatory cascade induced by transendothelial migration at the early stage of the IRI response, which could promptly remodel the immune microenvironment and attenuate the reperfusion injury.

### In vivo hepatic protection by CPTD in acute DILI

**Figure 7:**
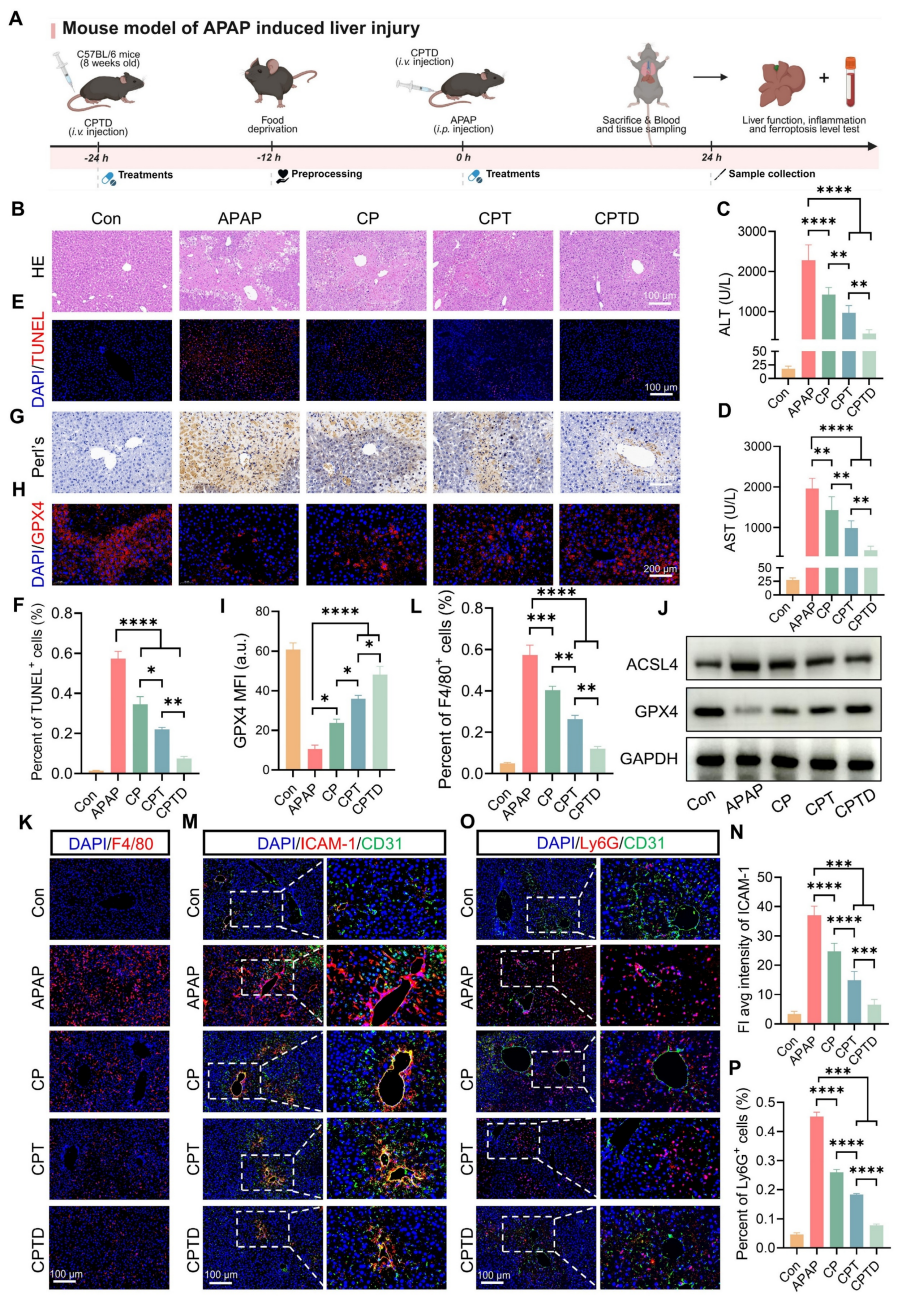
Therapeutic efficacy of CPTD in mouse model of APAP-induced ALI. (**A**) Schematic illustration for studying CPTD-mediated hepatocyte protection in the APAP-induced ALI mouse model. (**B**) Representative images of H&E staining of the liver. (**C-D**) Quantification of ALT and AST in serum of mice receiving or not receiving APAP following treatment with CP, CPT or CPTD (n = 6). (**E-F**) Representative images of TUNEL staining of liver sections (**E**) and quantitative statistics of the number of TUNEL-positive cells (**F**, n = 5). (**G**) Representative images of Prussian blue staining in liver sections (n = 5). (**H-I**) GPX4 fluorescence images of mouse liver sections (**H**) and quantification analysis (**I**) after treatment with various preparations (n = 5). (**J**) Western blot of ACSL4 and GPX4 in liver tissues of mice treated with different preparations (n = 3). (**K-L**) Representative images of F4/80 IF in liver sections (**K**) and percentage of F4/80 positive cells (**L**) of mice in each treatment group (n = 5). (**M-N**) Representative images of ICAM-1/CD31 fluorescence in liver sections (**M**) and quantification analysis of ICAM-1 fluorescence (**N**) of mice in each treatment group (n = 5). (**O-P**) Representative images of Ly6G/CD31 fluorescence in liver sections (**O**) and percentage of Ly6G positive cells (**P**) of mice in each treatment group (n = 5). Data are presented as mean ± SD, n.s. not significant, *p < 0.05, **p < 0.01, ***p < 0.001, and ****p < 0.0001 (one-way ANOVA with Tukey's post hoc test).

APAP overdose is the major cause of DILI, which often progresses to acute liver failure. To investigate whether CPTD provided the same protective effect against acute DILI, an intraperitoneal injection of excess APAP was used to simulate acute DILI. As shown in Figure 7A, the mice were intravenously administered a single dose of CPTD 24 h prior to APAP administration, followed by another intravenous injection of CPTD simultaneously with APAP administration. The extent of liver injury was assessed 24 h later. We observed severe hepatic injury on HE slides, with large areas of necrosis around the central vein accompanied by inflammatory cell infiltration, and significant elevations of ALT and AST indicating severe impairment of liver function (Figure 7B-D). However, CPTD reduced serum ALT and AST and decreased the extent of hepatic injury considerably, implying a protective effect of CPTD against APAP-induced ALI. Additionally, TUNEL staining supported this finding, with CPTD reducing 57.3% of TUNEL-positive cells due to APAP to 7.6%, which dramatically reduced necrotic areas of APAP-intoxicated livers and protected the liver structure (Figure 7E-F).

At safe doses, APAP is rapidly conjugated with glucuronic acid and sulfate, or metabolized through the hepatic cytochrome P450 pathway via GSH into non-toxic metabolites 42. Excessive APAP rapidly depletes GSH, and the accumulated toxic metabolic intermediate N-acetyl-p-benzoquinone imine (NAPQI) inhibits mitochondrial respiration and energy metabolism by binding to mitochondrial membrane proteins, leading to an imbalance in the mitochondrial antioxidant system 43. DHE staining showed that CPTD significantly ameliorated the accumulation of superoxide in the liver during APAP intoxication (Figure S22A-B, Supporting Information). We then further assessed the effect of CPTD on hepatic oxidative stress after APAP intoxication. Unsurprisingly, the CPTD formulation exhibited a stronger antioxidant effect, with a 66.1% decrease in MDA levels (Figure S23A, Supporting Information). Serum GSH and SOD levels increased to 17.67 ± 1.43 μg/mg protein and 53.27 ± 3.86 μg/mg protein, respectively (Figure S23B-C, Supporting Information). In this process, mitochondrial homeostasis was protected by the efficient antioxidant effect of CPTD multicomponents, which restored mitochondrial function and reduced hepatocyte death.

Moreover, ferroptosis features are usually observed in acute liver failure caused by APAP overdose. Prussian blue staining was observed to accompany large Fe³⁺ accumulation in the injured area, which was dramatically reduced by CPTD treatment (Figure 7G). The expression level of GPX4 was significantly downregulated following APAP-mediated hepatotoxicity, which is likely attributed to the formation of conjugates between excessively generated NAPQI and GSH (Figure 7H). The depletion of GSH disrupts redox homeostasis, thereby compromising the maintenance of GPX4. However, CPTD treatment can effectively ameliorate this condition, restoring GPX4 expression (Figure 7H-I). Besides, the down-regulation of ACSL4 expression and up-regulation of GPX4 expression reinforced the fact that CPTD was more advantageous in inhibiting ferroptosis during DILI compared to other combinations of preparations (Figure 7J; Figure S24A, Supporting information). Therefore, CPTD provides significant protection against APAP intoxication by alleviating ferroptosis in the liver, which largely depends on its ability to stabilize the intracellular LIP.

The progression of liver injury induced by APAP overdose involves a secondary inflammatory response promoted by the release of inflammatory mediators. NAPQI causes cellular protein damage and mediates hepatocellular necrosis, leading to the release of damage-associated molecular patterns (DAMPs) 44. DAMPs activate host cells to release injury mediators and chemokines, which further exacerbate hepatic inflammatory responses through the recruitment of inflammatory cells 45. Macrophage recruitment could be reduced by administration of CP and CPT, and it was exciting to note that CPTD was more effective in inhibiting macrophage recruitment in the presence of DILI, with almost negligible elevation of F4/80 expression (Figure 7K-L). The decrease in the inflammatory mediators IL-1β and IL-6 also indicated that CPTD performs the same function of inhibiting inflammatory overreaction in DILI (Figure S25A-B, Supporting information). Furthermore, the activation of inflammatory cascade signaling attracts circulating neutrophils to accumulate and become activated in the liver. Studies have demonstrated that depleting or inhibiting neutrophils can effectively alleviate APAP-induced ALI, likely due to reduced neutrophil infiltration diminishing the accumulation of damaging substances such as IL-1α and myeloperoxidase (MPO) 46,47. We observed that under the context of APAP-induced ALI, the liver exhibited marked sinusoidal endothelial injury, upregulation of ICAM-1 expression, and increased neutrophil infiltration (Figure 7M-P). In contrast, CPTD treatment induced significant microenvironmental remodeling in the mouse liver, manifested as a substantial reduction in both the extent of vascular endothelial injury and the proportion of neutrophils. APAP intoxication is an immune response typically considered to mediate the development of systemic inflammatory response syndrome (SIRS), which can trigger the severe consequences of multi-organ failure. Following APAP-induced injury, hepatocytes release substantial inflammatory mediators that collectively activate the NF-κB pathway, leading to the upregulation of pro-inflammatory cytokines (e.g., TNF-α, IL-1β, IL-6). These cytokines further stimulate NF-κB activation, establishing a self-amplifying regulatory loop. Equally critical, tissue inflammation, apoptosis, and necrosis activate the NLRP3 inflammasome, exacerbating the release of inflammatory factors. Our findings demonstrate that CPTD effectively mitigates hepatic inflammation post-APAP injury by significantly suppressing both NF-κB pathway activation and NLRP3 upregulation, thereby underscoring CPTD's potential to reduce the incidence of systemic inflammatory response syndrome (SIRS) following APAP damage (Figure S26A-C, Supporting Information). Collectively, CPTD can suppress hepatic inflammation induced by APAP intoxication. Current evidence suggests that CPTD likely suppresses perturbations at the interface between sinusoidal spaces and immune cells and effectively suppresses ferroptosis, making it an effective intervention for APAP overdose.

### Multi-mechanistic CPTD surpasses Ferrostatin-1 (Fer-1) in resolving ALI

**Figure 8:**
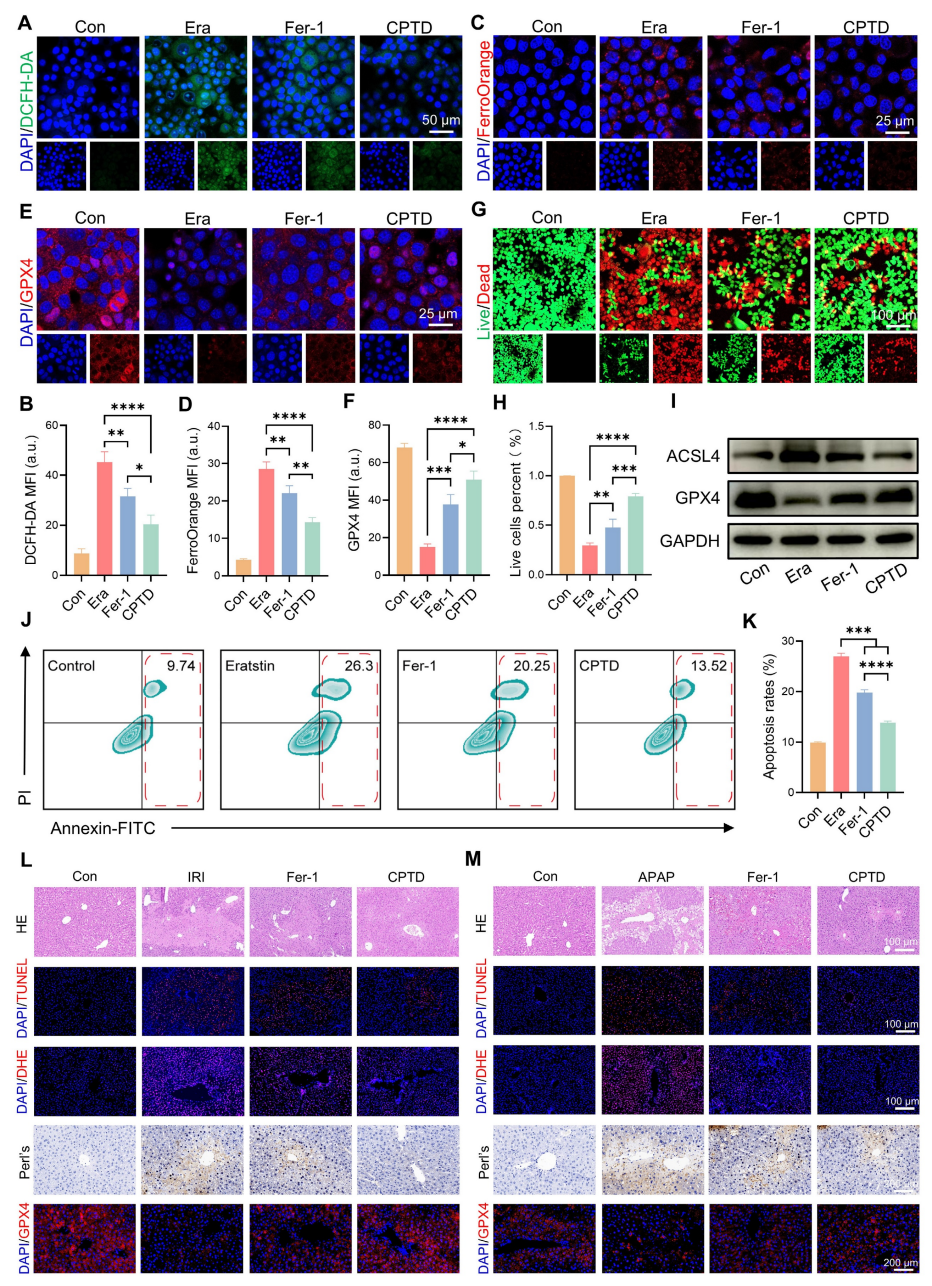
The therapeutic effect of CPTD is superior to that of Fer-1. (**A-B**) DCFH-DA staining (**A**) and quantitative analysis (**B**, n = 3) for ROS levels of AML12 cells pretreated with or without erastin stimulation following treatment with Fer-1 or CPTD. (**C-D**) FerroOrange staining for Fe^2+^ levels (**C**) and quantitative analysis (**D**, n = 3) of AML12 cells pretreated with or without erastin stimulation following treatment with Fer-1 or CPTD. (**E-F**) Expression levels and quantitative analysis of GPX4 by immunofluorescence. (**G-H**) Live/dead staining (**G**) and quantitative analysis (**H**, n = 3) in each treatment group. (**I**) Western blot of ACSL4 and GPX4 in AML12 cells treated with different preparations. (**J-K**) Flow cytometry (**J**) and quantitative analysis (**K**, n = 3) of apoptosis in each treatment group. (**L**) Representative images of HE, TUNEL, DHE, Prussian blue staining and GPX4 fluorescence in liver sections of HIRI mouse model in each treatment group. (**M**) Representative images of HE, TUNEL, DHE, Prussian blue staining and GPX4 fluorescence in liver sections of DILI mouse model in each treatment group. Data are presented as mean ± SD, n.s. not significant, *p < 0.05, **p < 0.01, ***p < 0.001, and ****p < 0.0001 (one-way ANOVA with Tukey's post hoc test).

Crosstalk between oxidative stress and ferroptosis amplifies their respective signaling pathways, exacerbating hepatocyte damage. Next, we investigated the differences in efficacy between CPTD and the commonly used ferroptosis inhibitor Fer-1 in the treatment of ALI to explore the clinical application potential of CPTD. Fer-1 is a synthetic antioxidant that prevents damage to membrane lipids through a reduction mechanism, thereby inhibiting ferroptosis. First, we evaluated the differences between CPTD and Fer-1 in modulating iron metabolism and protecting hepatocytes through in vitro experiments. Our findings demonstrate that CPTD exhibits significantly greater efficacy in alleviating Erastin-induced cellular damage. Specifically, CPTD substantially reduces ROS accumulation and effectively sequesters the LIP. In contrast, Fer-1 intervention showed inferior outcomes, manifested as elevated ROS levels and Fe²⁺ accumulation (Figure 8A-D). Similarly, CPTD treatment preserved significantly more GPX4 than Fer-1, suggesting superior maintenance of cellular redox homeostasis (Figure 8E-F). More crucially, the concomitant downregulation of ACSL4 and upregulation of GPX4 demonstrate that CPTD supplementation more effectively suppresses ferroptosis pathway activation than Fer-1 (Figure 8I; Figure S27A-B, Supporting information). Then, through live/dead staining, it was visually observed that CPTD could better protect AML12 cells (Figure 8G-H). The changes in apoptosis levels observed by flow cytometry further support our conclusions. Although both Fer-1 and CPTD treatment reduced cell death in AML12 cells, the degree of apoptosis was significantly lower in AML12 cells treated with CPTD (Figure 8J-K).

Subsequently, we further compared the efficacy differences between the two through in vivo experiments. We are delighted to report that CPTD treatment significantly surpassed Fer-1 in therapeutic efficacy against ALI, validated in both HIRI and DILI models (Figure 8L-M). Markedly reduced hepatocellular necrosis was evident in the CPTD-treated groups, whereas Fer-1-treated groups still exhibited severe hepatocyte necrosis and abundant TUNEL-positive cells (Figure 8L-M; Figure S28A and D, Supporting information). Furthermore, DHE staining confirmed that CPTD outperformed Fer-1 in suppressing cellular oxidative stress post-ALI (Figure 8L-M; Figure S28B and E, Supporting information). Diminished iron deposition indicated by Prussian blue staining demonstrated substantially improved iron metabolism in CPTD-treated groups. Ultimately, GPX4 expression dynamics confirmed that CPTD's multitarget therapeutic mechanism delivers significantly more potent suppression of ferroptosis and oxidative stress than Fer-1 (Figure 8L-M; Figure S28C and F, Supporting information). This compelling therapeutic efficacy demonstrates that CPTD, as a multi-targeted nanotuner, represents a promising therapeutic strategy for ALI with tremendous clinical potential.

## Discussion

How to efficiently reduce the accumulation of mROS and mLIP is the central theme of this study. Currently, there are few effective clinical treatment options for ALI, which particularly complicates the treatment of cases that rapidly progress to ALF. Navigating the complex mechanisms underlying the occurrence and progression of ALI presents formidable challenges for the development of effective treatment regimens. Therapeutic strategies aimed at reconstructing the antioxidant defense system can effectively alleviate ALI by suppressing excessive ROS production and the activation of inflammatory pathways. NAC, the only clinically used antioxidant detoxifier, improves oxidative stress by replenishing GSH to mitigate acute liver injury 48. However, limited clinical efficacy of NAC, coupled with the fact that some patients receiving recommended doses may still experience adverse reactions or progress to liver injury, highlights its therapeutic constraints 49. There is an urgent need to identify a universal therapeutic strategy to alleviate hepatocyte damage during ALI. As new studies continue to emerge, to the best of our knowledge, ferroptosis has been determined to be involved in the development of acute or chronic liver injuries 50. Our transcriptomic data analysis also supports the contribution of the activation of the ferroptosis pathway to ALI induced by two different causes. Therefore, therapeutic strategies targeting the inhibition of iron death hold great promise for mitigating ALI progression.

ALI is accompanied by hallmark features of cell death. In ALI induced by diverse stressors, preferential damage to the central venous (CV) zone of the hepatic lobule is commonly observed 51-53. This phenomenon may arise because hepatocytes in the CV zone receive comparatively fewer nutritional and metabolic supplies from peripheral blood than those in the portal vein (PV) zone and transition zone, rendering them more vulnerable to oxidative stress injury and iron metabolism dysregulation 54. The uncontrolled expansion of the LIP accelerates the initiation of ferroptosis programs in hepatocytes, serving as a critical driver of ALI. Under such pathological conditions, excessive iron accumulation is typically observed. The LIP is an intracellular, dynamically regulated iron reservoir, described as a putative cytosolic compartment enriched with chelatable, redox-active iron ions 55. Mitochondria are not only the primary sites of oxidative phosphorylation but also critical hubs for iron utilization and accumulation, essential for maintaining cellular homeostasis 14,56. During ALI, dysfunctional mitochondria are prone to generate excessive mROS, leading to mitochondrial rupture and impairing ATP production. Reducing mROS to restore mitochondrial function holds significant promise for ameliorating ALI. Mitochondrial iron is partially derived from the cellular LIP, with another portion originating from lysosomal reduction of Fe³⁺ and degradation of ferritin 31. In ALI, dysregulation of iron metabolism leads to excessive Fe²⁺ in the LIP, which catalyzes the explosive generation of ROS through the Fenton reaction. This inflicts a secondary hit on hepatocytes with pre-existing elevated oxidative stress, triggering membrane lipid peroxidation and exacerbating the progression of cell death.

Mitochondria-targeting approaches offer a promising therapeutic strategy for drug delivery in ALI. Functional nanomaterials, by addressing the limitations of existing therapies and enhancing their efficacy, present significant potential for the prevention and treatment of ALI. Compared to small-molecule compounds, synthetic nanomaterials demonstrate superior stability and highly efficient, sustained activity in scavenging multiple types of ROS 57. These advantages position artificial nanomaterials as promising non-pharmacological therapeutic approaches for disease treatment. Furthermore, the protection and restoration of mitochondrial function are critical aspects requiring focused attention. The mROS are primary contributors to mitochondrial damage; however, their limited diffusion radius (approximately 20 nm) poses a challenge for effectively targeting and scavenging ROS within mitochondrial regions, thereby limiting the capacity to preserve and restore mitochondrial function 58.

We have to admit the limitations of the current stage. First, current animal models employ mono-factorial ALI induction, which commonly used to simulate ALI, but the clinical realities in human patients involve intricate etiological factors and significant individual heterogeneity. Consequently, deploying animal disease models faces inherent challenges in effectively replicating complex clinical scenarios. Such difficulties remain pervasive in foundational research, where limited clinical relevance stems from the absence of human data. Thus, while our current findings offer mechanistic insights, future validation using ALI patient specimens is imperative to substantiate CPTD's promising therapeutic potential. Second, the introduction of metal-based nanomaterials notably raises safety concerns regarding prolonged hepatic retention. During ALI, severely compromised liver function coupled with the accumulation of nanoparticles in hepatic tissue creates potential risks for extended nanomaterial persistence. Optimizing therapeutic dosing to achieve sustained efficacy while ensuring progressive degradation to mitigate safety hazards associated with prolonged retention represents a pressing challenge in current ALI nanotherapy development. Third, the challenge of immune system interactions: The administration of nanomedicines in humans inherently triggers activation of the innate immune system. Consequently, understanding the reciprocal impacts between the immune system and nanomedicine transport *in vivo*, along with the potential for leveraging nanotherapeutics to modulate immune responses, will emerge as focal points for clinical translation.

In our study, CPTD exhibits exceptional SOD- and CAT-like enzymatic activities, and its TPP modification enables selective accumulation within mitochondria. Upon entering hepatocytes, CPTD effectively localizes around mitochondria and responsively releases DFO, which simultaneously reduces ROS accumulation and inhibits excessive LIP expansion. We validated the therapeutic efficacy of CPTD in vitro and in two distinct ALI models, demonstrating its remarkable ability to maintain redox homeostasis and LIP equilibrium. Furthermore, CPTD effectively suppresses the activation of ferroptosis pathways, thereby reducing liver injury, alleviating oxidative stress and inhibiting ferroptosis.

## Conclusion

Here, we developed CPTD, a multifunctional nanotuner for hepatoprotection, which effectively localizes around mitochondria and stabilized the oxidation and iron homeostasis. By harnessing its diverse bio-antioxidant enzymatic activities, CPTD suppresses ROS bursts through ROS scavenging and spatiotemporally modulates the LIP via targeted iron chelation, thereby preventing ferroptosis activation. Importantly, CPTD improves mitochondrial function by eliminating mROS and mLIP. In murine models of HIRI and APAP-induced ALI, CPTD efficiently accumulates in the liver, exerting potent antioxidant and anti-ferroptosis effects. Furthermore, CPTD reduces inflammatory mediator release and immune cell recruitment in the injury microenvironment, ultimately restoring hepatic function. In summary, we synthesized a multifunctional nanotuner integrating mitochondrial targeting, antioxidant activity, and iron chelation, which demonstrates remarkable therapeutic efficacy both in vitro and in vivo. This dual-pronged therapeutic strategy provides an innovative approach for the clinical management of ALI.

## Methods and Materials

### Materials

Ce(NO_3_)_3_ · 6H_2_O (Aladdin, C105378), Na_3_PO_4_ (Macklin, S818159), PVP (Mw 10000, Macklin, P816209), Tris-HCl (Solarbio, T8230), NaOH (Macklin, S817977), Dopamine hydrochloride (Macklin, D756911), 4-arm-PEG-NH_2_ (Macklin, P971414), Sulfo-NHS (ThermoFisher, 24510), EDC·HCl (Aladdin, E106172), TPP (Macklin, C805851), DFO (Macklin, B885980), Erastin (MCE, HY-15763), FerroOrange (MCE, HY-D1913), 4′, 6-diamidino-2-phenylindole (DAPI, Beyotime, C1002), DCFH-DA (Beyotime, S0033S), BODIPY 581/591 C11 (Beyotime, S0043S), MitoSOX Red (Beyotime, S0061S), JC-1 probe (Beyotime, C2003S), Calcein/Pl probe (Beyotime, C2015M), Mitotracker green (Beyotime, C1048), BCA protein assay kit (Solarbio, PC0020), Cell Counting Kit-8 (CCK-8, Meilun Biotechnology, MA0218), AnnexinV-FITC/PI apoptosis kit (Meilun Biotechnology, MA0220-1), MitoFerroGreen (Dojindo Laboratories, M489), CY5.5-NHS (Ruixibio), anti-GPX4 (Proteintech, 30388-1-AP), anti-ACSL4 (Abcam, ab155282), anti-GAPDH (Proteintech, 60004-1-Ig), anti-F4/80 (Proteintech, 29414-1-AP), anti-CD31 (Proteintech, 28083-1-AP), anti-ICAM-1 (Proteintech, 82827-8-RR), anti-Ly6G (CST, #87048).

### Bulk transcriptome data analysis

High-throughput sequencing expression profiling dataset GSE113024 for HIRI was downloaded from the GEO database (https: //www.ncbi.nlm.nih.gov/geo/). We included routinely preserved samples (n = 3) and post-reperfusion samples from liver transplantation (n = 3) in the follow-up study. The APAP-induced ALI cohort was derived from the study by Yu et al. which included 3 APAP-induced ALI samples and 3 control samples. Standard processing pipelines, including gene ID conversion, expression data normalization and PCA, were performed separately for each cohort to ensure the reliability of data analysis. The *limma* package was used for between-group difference analysis, and *P* < 0.05 was considered DEGs. The *Bioenrichr* and *ClusterProfiler* packages were used for subsequent KEGG enrichment analysis based on DEGs and *P* < 0.05 was considered statistically different. The *ggplot* R package was used for graph visualization.

### Animals and research compliances

8-week-old C57 BL/6 mice were purchased from the Laboratory Animal Center of Zhengzhou University (Zhengzhou, China). Mice were housed in captivity in a constant environment with a light/dark cycle of 12 h each, temperature of 20-25 °C, and humidity of 40-60%. Mice are provided with free access to water and food, and the rearing facility is free of specific pathogens. Animal experiments were performed following the ethical guidelines approved by the Ethics Committee of the First Affiliated Hospital of Zhengzhou University.

### Cell lines

Mouse normal hepatocytes (AML12 cells, RRID: CVCL_0140) were purchased from Pricella Life Sciences Ltd (Wuhan, China). The cell culture medium used Dulbecco's Modified Eagle Medium/Nutrient Mixture F-12 (DMEM/F12, Gibco) supplemented with 10% FBS (fetal bovine serum, Gibco), 1% penicillin/streptomycin (Gibco), 0.5% ITS-G (Insulin-Transferrin-Selenium, 100 ×, MCE, HY-150287) and 40 ng/mL dexamethasone (MCE, HY-14648), and was cultured in a humidified incubator at a constant 37 °C with 5% CO₂.

### In vitro hepatocyte injury model

*H/R cell Model.* The AML12 cells H/R model was employed to simulate HIRI in vitro. Briefly, when the cell density reached approximately 80%, the medium was replaced with a sugar-free and serum-free medium, and the cells were transferred to a tri-gas incubator (5% CO₂, 94% N₂, and 1% O₂) for hypoxic culture for 12 h. After hypoxia, the normal medium was replaced, and the cells were reoxygenated in a normoxic incubator for 6 h.

*APAP cell injury model.* APAP was fully dissolved in PBS at 37 °C to make a 100 μM/mL stock solution. When AML12 cells were cultured to nearly 80% density, cells were incubated for 6h using complete medium containing APAP (10 μM/mL).

*Ferroptosis cell model.* AML12 cells were inoculated into 6-well plates, 96-well plates, or 2 cm confocal dishes with cell inoculation quantities of 1 × 10^6^ per well, 1 × 10^5^ per well and 5 × 10^6^ per well, respectively, unless otherwise specified. After the apposition was stabilized, the model of acute hepatocyte injury by ferroptosis was established through treatment with 10 μM/mL erastin. The effect of drug treatment was further assessed by co-incubation with CP, CPT or CPTD (10μg/mL) for 12 h.

*Cell Protection Evaluation*. To compare the anti-apoptotic effects of CPTD and Fer-1 in Erastin-induced cells, an acute hepatocyte injury model was established using erastin. Cells were then co-incubated with or without free CPTD (10 μg/mL) or Fer-1 (1 μΜ/L, HY-100579, MCE, USA) for 12 h. For Fer-1, pre-treat with Fer-1 for 1 h before treating with erastin. Subsequent experiments were conducted to evaluate the protective efficacy of CPTD and Fer-1 on AML12 cells.

### Synthesis of CN, CP, CPT, CPTD

First, 0.434 g of Ce(NO₃)₃·6H₂O, 0.003 g of Na₃PO₄, and 0.1 g of PVP were dissolved in 40 mL of deionized water and then transferred into a high-pressure reactor. The reaction mixture was reacted at 170 ℃ for 12 h. After the reaction was completed, the nanoparticles were separated by centrifugation at 12,000 rpm and washed three times with deionized water to obtain CN.

For the synthesis of CP, 5 mL of CN (2 mg/mL) was added to 25 mL of deionized water, followed by the sequential addition of 0.03 g of Tri-HCl and 150 µL of NaOH (40 mg/mL). After mixing, 15 mg of dopamine hydrochloride was added, and the reaction mixture was rapidly stirred at room temperature for 30 min. The product was then purified by centrifugation at 12,000 rpm to obtain CP.

To modify the surface of CP with PEG and TPP, 1 mL of CP (containing 1 mg of CN) was mixed with 2 mg of 4-arm PEG-NH₂ and stirred for 2 h. For the activation of TPP, 42 µL of TPP (10 mg/mL), 2 mg of EDC·HCl, and 2 mg of Sulfo-NHS were added to 1 mL of water and stirred at room temperature for 2 h. Then, 200 µL of the reaction mixture was added to the above CP solution and stirred overnight. The product was purified by centrifugation at 12,000 rpm and washed three times with deionized water to obtain CPT.

Finally, to load DFO onto the surface of CPT, 1 mL of CPT solution (with a CN concentration of 1 mg/mL) was mixed with 1 mL of DFO solution (1 mg/mL) and stirred vigorously overnight. The excess DFO was removed by centrifugation at 12,000 rpm to obtain CPTD. To establish the DFO calibration curve, we reacted excess FeCl₃ (10 mg/mL) with DFO and recorded its absorption spectrum between 400-800 nm, observing a distinct peak at 420 nm. Subsequently, the absorbance at this wavelength was measured at different DFO concentrations, and a standard curve was plotted based on the linear correlation between the absorbance and the concentration of the DFO/Fe³⁺ complex. For quantifying DFO release from CPTD, nanoparticles were incubated with H₂O₂ (2 mM) for specified durations, followed by centrifugation at 12,000 rpm to separate CPTD from released DFO. The supernatant was reacted with Fe³⁺, and the absorbance of the resulting complex at 420 nm was measured and interpolated against the standard curve to determine DFO concentration. The loading efficiency (LE) and encapsulation efficiency (EE) of DFO were determined by measuring the absorbance at 430 nm after reacting the residual DFO in the supernatant with an excess of FeCl₃ solution. The specific calculation formulas are as follows: LE = weight of DFO in CPTD / weight of CPTD × 100%, EE = weight of DFO in CPTD / weight of total DFO × 100%.

### Characterization of CPTD

XRD (PANalytical X'pert3) was used to evaluate the crystal structure of CN and to determine the position of CN diffraction peaks concerning a standard card. Determination of elemental composition and valence distribution in CN using XPS (K-alpha, Thermo Fisher). The morphology, particle size distribution and elemental mapping of the nanoparticles were observed using TEM (JEOL F200, Japan) at an accelerating voltage of 120 kV. The TGA curves of different samples were recorded from room temperature to 1200 °C using a simultaneous thermal analyzer (TGA-DSC, Mettler Toledo). DLS measurements were performed using a Zetasizer Nano ZS (Malvern Panalytical) to determine the hydrated particle size of the nanoparticles as well as the zeta potential. And the dispersibility of nanoparticles in different solvents was evaluated by the Polydispersity Index (PDI). The successful stepwise synthesis of CPTD was confirmed through UV-Vis (Lambda 950, U.S.) and FTIR (Nicolet iS50 FTIR, Thermo Fisher) spectroscopic analyses.

### Evaluation of ROS and free radicals scavenging capacity of CPTD

H_2_O_2_ scavenging capacity of CPTD was measured by Hydrogen Peroxide Assay Kit (Beyotime, S0038). Briefly, different concentrations of CPTD (0 μg/mL, 6.25 μg/mL, 12.5 μg/mL, 25 μg/mL, 50 μg/mL, 100 μg/mL) were incubated with H₂O₂ at room temperature for 1 h, and the remaining H₂O₂ was quantified by measuring the absorption spectrum of the samples at 540-570 nm using a spectrophotometer. The ·O_2_^-^ scavenging capacity of CPTD was evaluated by measuring the absorption spectrum at 530 nm using the Superoxide Anion Content Assay Kit (Boxbio, AKAO008C) according to the manufacturer's procedure. The Hydroxyl Radical Scavenging Capacity Assay Kit (Elabscience, E-BC-K527-M) was used to determine the ·OH scavenging capacity of CPTD. The absorption spectrum at 510 nm was measured according to the manufacturer's protocol.

In addition, the antioxidant capacity of CPTD was further determined by DPPH and 2,2'-azino-bis (3-ethylbenzthiazoline-6-sulfonic acid) (ABTS). Specifically, in the DPPH assay, varying concentrations of CPTD (0 μg/mL, 6.25 μg/mL, 12.5 μg/mL, 25 μg/mL, 50 μg/mL, 100 μg/mL) were introduced into the DPPH working solution, and the absorption spectra at 515 nm were detected using UV-Vis spectroscopy. For the ABTS scavenging assay, different concentrations of CPTD were similarly added to the ABTS working solution, reacted at room temperature in the dark for 6 min, and the absorbance at 405 nm was measured via UV-Vis spectroscopy.

### Measurement of CPTD iron chelating ability

To confirm the iron chelating properties of CPTD retaining DFO, the iron chelating capacity of CPTD was determined using Ferrous Ion Content Assay Kit (Solarbio, BC5415), Cell Iron Content Assay Kit (Solarbio, BC5315). The iron chelating capacity of nanoparticles with different components (CN, CP, CPT, CPTD) was measured to evaluate the superiority of multi-component CPTD. Furthermore, CPTD demonstrated dose-dependent iron chelation ability at varying concentrations (0 μg/mL, 6.25 μg/mL, 12.5 μg/mL, 25 μg/mL, 50 μg/mL, 100 μg/mL). In brief, according to the manufacturer's instructions, nanoparticles were incubated with a working solution containing FeSO₄ for 10 min, and the absorbance at 593 nm was measured to determine Fe²⁺ concentration. Separately, nanoparticles were co-incubated with a working solution containing hydroxylamine hydrochloride for 10 min, and the absorbance at 510 nm was measured to determine Fe^3+^ concentration.

### Cell viability assay, in vitro cytotoxicity assay

CCK-8 and AM/PI staining assay were used to detect the effect of CPTD treatment on cell viability. For CCK-8 experiments, AML12 cells were inoculated in 96-well plates to make them adhere to the wall and incubated for 24 h. The cells were incubated for 24 h and 48 h using media containing different concentrations of CPTD (0 μg/mL, 6.25 μg/mL, 12.5 μg/mL, 25 μg/mL, 50 μg/mL, 100 μg/mL). Then incubate for 1 h at 37 ℃ using medium containing 10% CCK-8 solution. The absorbance at 450 nm of each treatment group was subsequently measured using microplate reader (Synergy H1, BioTek) according to the manufacturer's protocol. For AM/PI staining, AML12 cells were inoculated at 5 × 10^5^ per well and cultured overnight in 2 cm confocal dishes. After treating the cells with the same concentration gradient of CPTD for 48 h, AM/PI staining was done according to the reagent vendor's instructions. Finally, cell viability was observed in real time under CLSM (Leica SP8 STED 3X, Germany).

### Cellular uptake and mitochondrial targeting assays

*Cellular uptake*. The efficiency of nanoparticle uptake by AML12 cells was investigated using Cy5.5-labeled nanoparticles (CP, CPT and CPTD). Briefly, Cy5.5-NHS (1mg/mL, 10μL) was mixed with CPTD (1mg/mL, 1 mL) and stirred at room temperature away from light for 12 h. Then, unreacted Cy5.5 was removed by ultrafiltration. Cy5.5-labeled CP and CPT were prepared as above. Cy5.5-labeled CP, CPT and CPTD were incubated with AML12 cells for 4 h, respectively. The fluorescence intensity and the localization of CPTD within the cells were observed in real time using CLSM. For flow cytometry-based assays, AML12 cells were collected at determined time points (0 h, 1 h, 2 h, 4 h) and resuspended in PBS, and 10,000 cells per group were randomly captured to quantify fluorescence intensity by ImageJ software.

*Mitochondrial co-localization analysis*. Briefly, cells were inoculated in 2 cm confocal dishes and stabilized for 24 h to make them firmly attached to the surface. Cell nuclei were labeled using Hoechst (blue), mitochondria were labeled using Mitotracker (green), and then Cy5.5-labeled CPTD was added which was then imaged using CLSM at specific times. The mitochondrial targeting ability of CPTD was assessed by calculating Pearson correlation coefficients for Cy5.5 and Mitotracker fluorescence intensities.

### ROS, intracellular iron, and LPO measurement

*ROS measurement.* AML12 cells were harvested after drug treatment and immediately co-incubated with DCFH-DA probe (10 μM/mL) for 20 min at 37 °C for ROS evaluation. Mitochondrial reactive oxygen species were evaluated by MitoSox probes. Intramitochondrial superoxide reacts with Mitosox to produce red fluorescence. MitoSox working solution is configured from 1 µl MitoSOX Red (5 mM) and 1 ml PBS. Cells were stained by co-incubation with MitoSOX working solution at 37 ºC for 20 min. Besides, the mitochondrial membrane potential of cells in each treatment group was detected using the JC-1 probe. When the mitochondrial membrane potential is high, JC-1 can easily cross the mitochondrial membrane and accumulate as J-aggregates (red fluorescent) in the mitochondria; on the contrary, the decrease or disappearance of the mitochondrial membrane potential prevents the entry of JC-1 into the cell so that it exists in the form of green fluorescent monomers. Specifically, cells were stained by incubation with 10 µg/mL JC-1 at 37 °C for 20 min.

*Intracellular iron measurement*. Intracellular Fe^2+^ assay was performed with a FerroOrange probe (red fluorescence). FerroOrange was diluted to a final concentration of 1 μM/mL FerroOrange working solution according to the manufacturer's instructions. The washed cells were stained by co-incubation with FerroOrange working solution at 37 °C for 30 min. Determination of intra-mitochondrial iron using the Mito-FerroGreen probe. Briefly, HBSS buffer was diluted with the Mito-FerroGreen storage solution to make a concentration of 5 μM/mL of Mito-FerroGreen working solution. Subsequently, the washed cells were co-incubated with the Mito-FerroGreen working solution at 37 °C for 30 min to complete the staining. Intracellular Fe^2+^ concentration and total Fe concentration were quantified using the Ferrous Ion Content Assay Kit and Cell Iron Content Assay Kit, respectively. The samples were processed according to the instructions on the manufacture, the absorbance at specific wavelengths (593 nm and 510 nm) was obtained using a microplate reader and the concentration was determined.

*LPO measurement.* To analyze LPO levels, BODIPY 581/591 C11 was diluted at a ratio of 1:1000 to configure a working solution with a final concentration of 2 μM/mL. Staining was accomplished by co-incubating the washed cells with BODIPY 581/591 C11 working solution at 37 °C for 20 min.

Fluorescence images were obtained using CLSM observation. For flow cytometry-based assays, PBS resuspended AML12 cells were used, followed by up-loading to detect the fluorescence intensity of DCF and FerroOrange.

### Apoptosis assays

Cells were inoculated in six-well plates one day in advance to allow for adhesion. After 12 h of drug treatment, cells were digested using 0.25% trypsin and collected. Cells were washed using pre-cooled PBS and then stained for annexin V and PI according to the experimental procedure outlined by the reagent vendor. The treated samples were subjected to apoptosis analysis by BD Accuri™ C6 Plus (BD Biosciences) flow cytometer.

### In vivo biodistribution assay

For Cy5.5-labeled CPTD, CPTD (1 mg/ml) was stirred with Cy5.5 at a mass ratio of 100:1 in deionized water at room temperature overnight. Then, Cy5.5-labeled CPTD was obtained by centrifugation at 1000 rpm and resuspended in saline before injection.

The mice were randomly divided into three groups. Cy5.5-labeled CPTD (1.5 mg/kg) was administered to mice via tail vein injection; the control group received equal amounts of Cy5.5, and the blank control received a saline injection. The in vivo distribution of CPTD was recorded using a multifunctional fluorescence imaging system (PerkinElmer IVIS spectrum, U.S.) at 3 h, 6 h, 12 h, and 24 h, respectively. Mice were euthanized at 24 h to obtain the major organs (heart, liver, spleen, lung, kidney), and the intensity of fluorescence distribution in each organ was assessed.

### Biocompatibility analysis

To evaluate the biocompatibility of CPTD, intravenous injection of therapeutic dose of drug at 1.5 mg/kg (calculated as CeO₂), and the major organs (heart, liver, spleen, lung, and kidney) of each group of mice were removed for HE staining after 24 h to check for histopathological changes. In addition, serum was collected from each group of mice and the levels of ALT, AST, creatinine, BUN, LDH, and CK were detected using kits according to the manufacturer's instructions. Furthermore, to evaluate whether CPTD exhibits delayed-onset toxicity, we administered a therapeutic dose of CPTD intravenously on each of the first three days. The injury of major organs was assessed at different time points (days 1, 3, 5, 7 and 14) post-administration.

### Establishment of ALI mouse model

*IRI-induced ALI mouse model.* Mice were fasted for 12 h before surgery. Mice were anesthetized using an intraperitoneal injection of 20 μL/g Delivector™ Avertin (Shanghai Dowobio Biotechnology, DW3100). Midline dissection was performed to open the layers of the abdominal wall of the mice layer by layer, and the periportal tissues were carefully freed to expose the hepatic tip controlling blood flow in the left outer and middle lobes. The hepatic tip was then clamped using noninvasive vascular clips to cause ischemia and timed for 90 min. During this period, saline-moistened sterile gauze was used to cover the abdominal wall and the mice were placed on a 37 °C thermostatic plate to avoid temperature loss. At the end of the ischemic period, the clamps were released to allow reperfusion for 6 h, followed by suturing of the abdominal wall layers layer by layer. The sham-operated group was operated in the same way, except that the hepatic tips were not clamped. Animals were sacrificed and tissues were isolated for further analysis.

*APAP-induced ALI mouse model.* APAP (Aladdin, 103-90-2) was dissolved in sterile PBS to a concentration of 10 mg/ml. Mice were fasted for 12 h prior to APAP administration, and ALI was induced by intraperitoneal injection of 300 mg/kg APAP solution. After 24 h, mice were sacrificed and tissues were isolated for further analysis.

*Timing of systemic drug administration.* Take CPTD as an example, CPTD was pre-administered once via the tail vein 24 h prior to the establishment of the ALI mouse model. For the IRI model, the CPTD was given once again 30 min before blocking blood flow. In contrast, the APAP model was given a single dose of CPTD at the same time as APAP intoxication. The dose of each drug in the treatment group was administered at 1.5 mg/kg (in terms of CeO_2_), and the same dose of saline was given in the control and model groups.

### Liver function measurement

Blood samples were collected from mice via the inferior vena cava and centrifuged at 3000 g for 5 min. The supernatant was then collected to measure ALT and AST levels, as per the manufacturer's instructions (JianChen Bioengineering Institute, Nanjing, China). Take fresh liver tissue, wash, grind, and resuspend it, then measure the levels of GSH, MAD, and SOD (JianChen Bioengineering Institute, Nanjing, China) in the tissue homogenate.

*H&E staining.* Fresh liver tissues were fixed in 10% formalin solution for 48 h, followed by paraffin embedding. The paraffin blocks were sectioned into 5-μm-thick slices. After hematoxylin and eosin (H&E) staining, histopathological changes were observed under an optical microscope.

*DHE staining.* Levels of superoxide in tissues were detected by dihydroethidium (DHE) fluorescent probe (Biosharp, Hefei, China). Briefly, fresh tissues were embedded in OCT and sectioned into 10 μm-thick slices. The sections were stained with 10 μM DHE in the dark for 20 min, followed by three washes with PBS. Nuclei were counterstained with DAPI, and the sections were then imaged.

*TUNEL staining.* Paraffin sections were incubated with TUNEL staining solution for 10 min at 37 °C, followed by washing three times using PBS. DAPI labeled nuclei were used to observe apoptosis levels using fluorescence microscopy.

### Prussian blue staining

Thoroughly dewax and hydrate the aforementioned paraffin sections. Then, according to the manufacturer's instructions, incubate the sections with Perls' staining working solution (Solarbio, Beijing, China) for 20 min. After incubation, rinse three times with ultrapure water. Dehydrate through a graded ethanol series, clear with xylene, and mount with neutral resin. Capture images under an optical microscope.

### Western blot

After treatment of cells or liver tissue with RIPA lysis buffer (Solarbio, Beijing, China) to extract total proteins, the protein concentration was quantified using a BCA kit (Solarbio, Beijing, China). Subsequently, the proteins were mixed with loading buffer and boiled for 5 min. Each sample (20 μg per lane) was then loaded onto SDS-PAGE gels. After electrophoresis, proteins were transferred to PVDF membranes. The membranes were blocked with 5% skim milk at room temperature for 1 h, incubated with primary antibodies at 4 °C overnight, and then incubated with goat anti-rabbit or goat anti-mouse secondary antibodies at room temperature for 1 h. Protein bands were visualized under a ImageQuant™ (Cytiva, America) gel imaging system using ECL luminescent solution (NCM Biotech, Suzhou, China) and ImageJ software was used for quantification.

### Immunofluorescence

For cells cultured in confocal dishes, the medium was aspirated and washed twice with PBS, and the cells were fixed in 4% paraformaldehyde for 10 min. Cell permeabilization was treated with 0.1% Triton X-100 solution for 8 min. Cells were closed using 3% BSA to reduce non-specific binding. Cells were then incubated with GPX4 primary antibody (1:200) overnight at 4 °C. The cells were incubated with CoraLite® Plus 594-labeled goat anti-rabbit secondary antibody for 2 h at 37 °C. Fresh liver tissues were fixed with 4% paraformaldehyde, dehydrated step and then embedded using OCT, frozen and sectioned. The frozen sections were closed and incubated with primary antibody (including F4/80 (1:200), CD 31 (1:400), ICAM-1 (1:200) and Ly6G (1:100)) at 4 °C overnight. After washing, co-incubate with the secondary antibody for 2 h at room temperature and away from light. Sections were imaged using an inverted fluorescence microscope.

### Enzyme-Linked Immunosorbent Assay (ELISA)

Whole blood was taken from mice and allowed to clot for 30 min at room temperature, then centrifuged at 1000 g for 15 min to remove the supernatant for immediate detection. Mouse serum levels of IL-1β and IL-6 were measured using ELISA kits according to the manufacturer's procedure (Proteintech, Wuhan, China).

### Statistical analysis

Bioinformatics analysis was performed using R software (version 4.3.1), while data analysis and graphical visualization were conducted using GraphPad Prism 9. Data are expressed as mean ± SD. One-way analysis of variance (ANOVA) and Turkey's post hoc tests were used to compare differences among multiple groups. *P* < 0.05 is considered statistically significant.

## Supplementary Material

Supplementary figures.
