# Supplementary material for: Nanotuner targeting mitochondrial redox and iron homeostasis imbalance for the treatment of acute liver injury
Source: Theranostics. 2025 Aug 16;15(17):9131–58. doi: 10.7150/thno.119357 (PMC12439336; doi:10.7150/thno.119357)
Supplement: Supplementary file 1 — Supplementary figures. [file thnov15p9131s1.pdf]

Supporting Information

Nanotuner targeting mitochondrial redox and iron homeostasis imbalance for the treatment of acute liver injury

Minghao Li1,2†, Qiwei Yang3†, Jie Gao1,2, Xudong Liu1,2, Jihua Shi1,2, Wenzhi Guo1,2, Yi Zhang3, Qiwen Yu4, Xinzhi Sun3*, Shuijun Zhang1,2**

** Corresponding Author: Shuijun Zhang, zhangshuijun@zzu.edu.cn; Xinzhi Sun, sunxinzhi2023@163.com; Qiwen Yu, yuqiwen926@163.com*

†These authors contributed equally to this work

1Department of Hepatobiliary and Pancreatic Surgery, Henan Key Laboratory of Digestive Organ Transplantation, The First Affiliated Hospital of Zhengzhou University, Zhengzhou, China

2Zhengzhou Key Laboratory for Hepatobiliary & Pancreatic Diseases and Organ Transplantation, Zhengzhou, China

3Department of Orthopedics, The First Affiliated Hospital of Zhengzhou University, Zhengzhou, China

4Department of Surgery, The First Affiliated Hospital of Zhengzhou University, Zhengzhou, China.

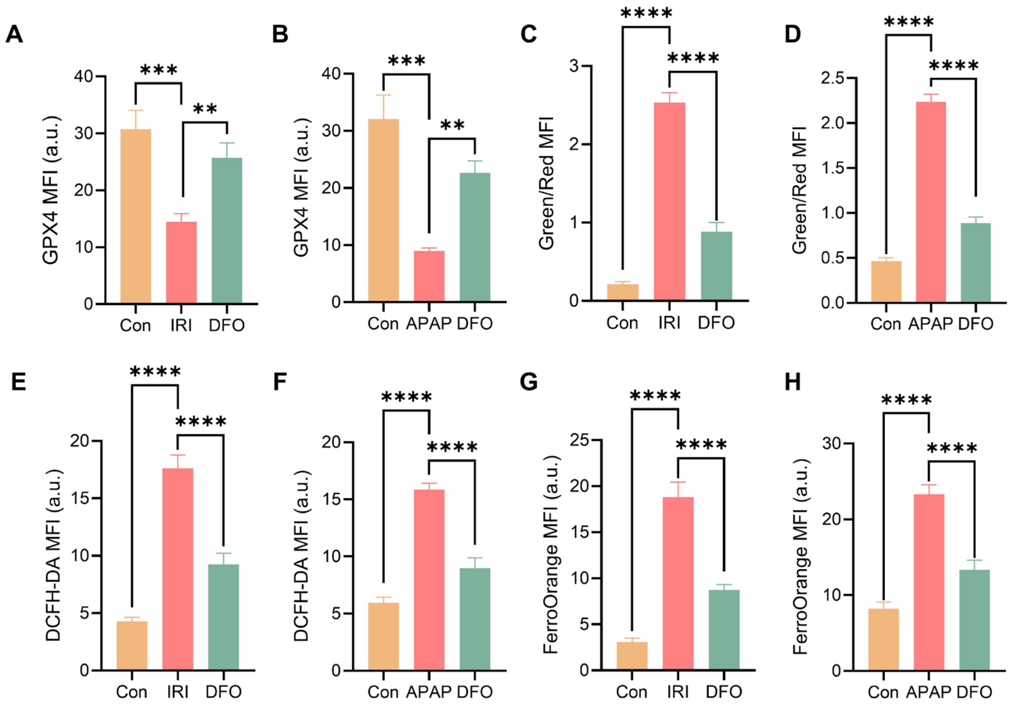

Figure S1. (**A-B**) Quantitative assessment of GPX4 fluorescence intensity in different cell groups (n = 3). (**C-D**) Quantitative assessment of the extent of LPO in the different cell groups, as measured by the green/red fluorescence ratio (n = 3). (**E-F**) Statistical analysis of DCFH-DA fluorescence intensities in different cell groups (n = 3). (**G-H**) Statistical analysis of FerroOrange fluorescence intensities in different cell groups (n = 3). Data are presented as mean ± SD, n.s. not significant, *p < 0.05, **p < 0.01, ***p < 0.001, and ****p < 0.0001 (one-way ANOVA with Tukey's post hoc test).

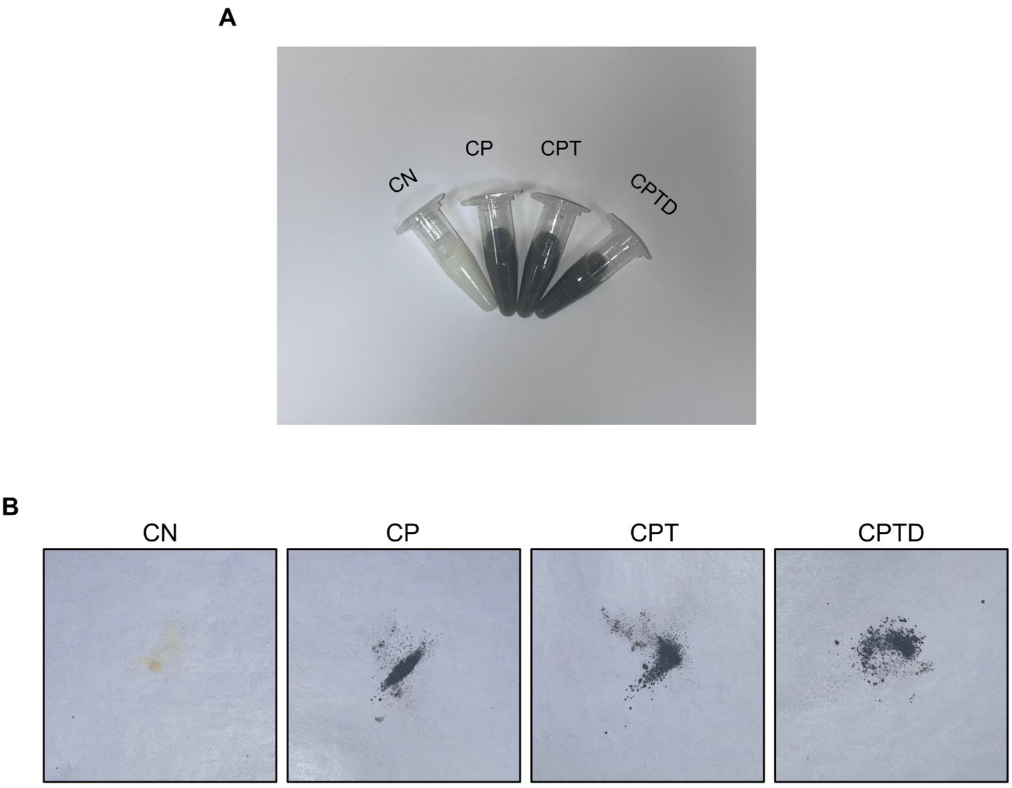

Figure S2. (**A**) Photographs of each group, including CN, CP, CPT and CPTD. (**B**) Photographs of each group after freeze-drying, including CN, CP, CPT and CPTD.

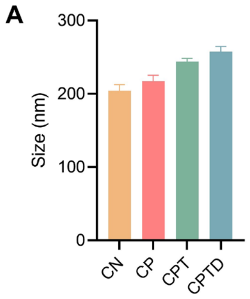

Figure S3. (**A**) Hydrodynamic particle sizes of CN, CP, CPT and CPTD (n = 3). Data are presented as mean ± SD.

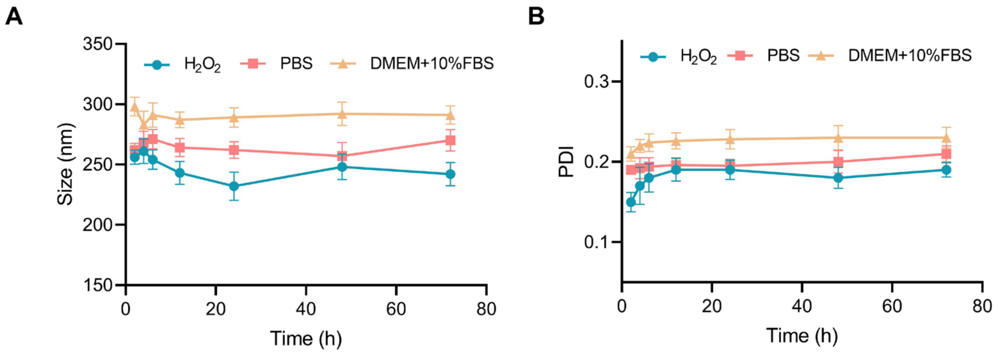

Figure S4. (**A-B**) DLS-measured particle size (**A**) and PDI (**B**) of CPTD stored in different solvents (n = 3). Data are presented as mean ± SD.

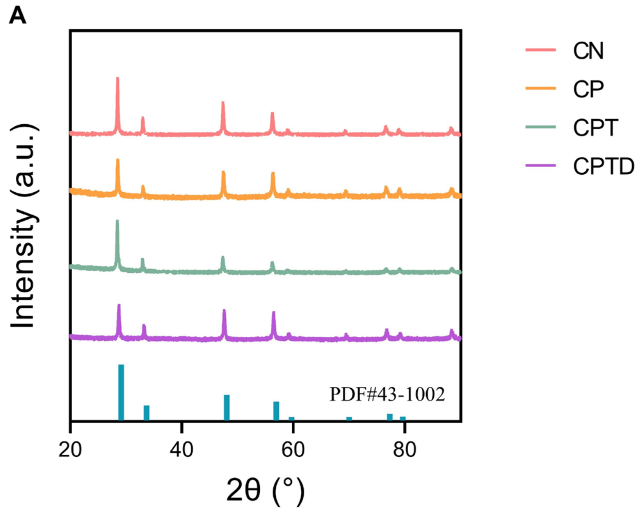

Figure S5. (**A**) XRD spectrum of CN, CP, CPT, CPTD and CeO2 standard card (PDF#43-1002).

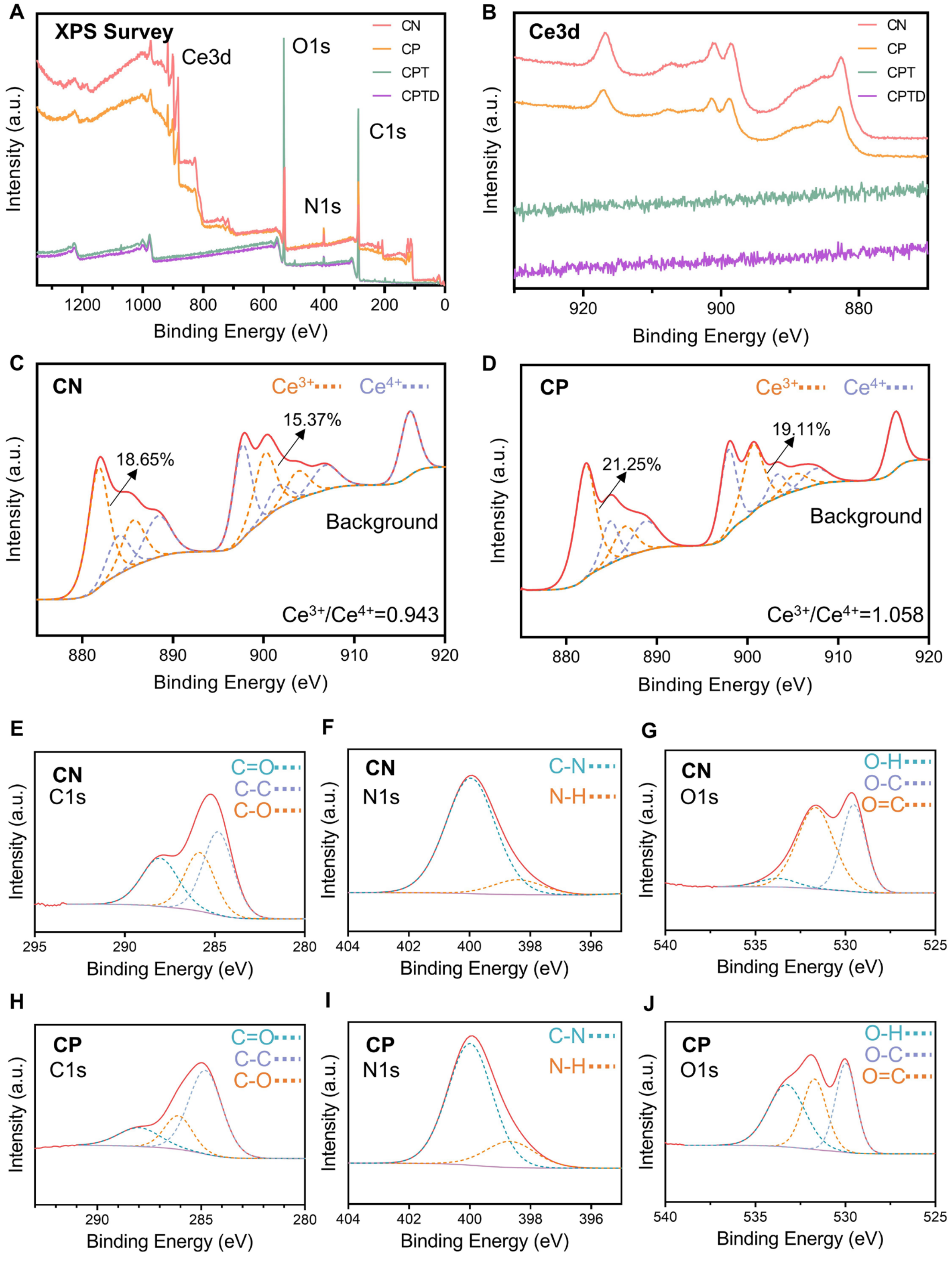

Figure S6. (**A**) Elemental composition of CN, CP, CPT and CPTD revealed by XPS. (**B**) Ce3d curves of CN, CP, CPT and CPTD revealed by XPS. (**C-D**) Ratio of Ce^{3+} to Ce^{4+} in CN (**C**) and CP (**D**) as revealed by peak fitting of Ce 3d. (**E-J**) XPS spectra of C1s, N1s, and O1s for CN (**E-G**) and CP (**H-J**).

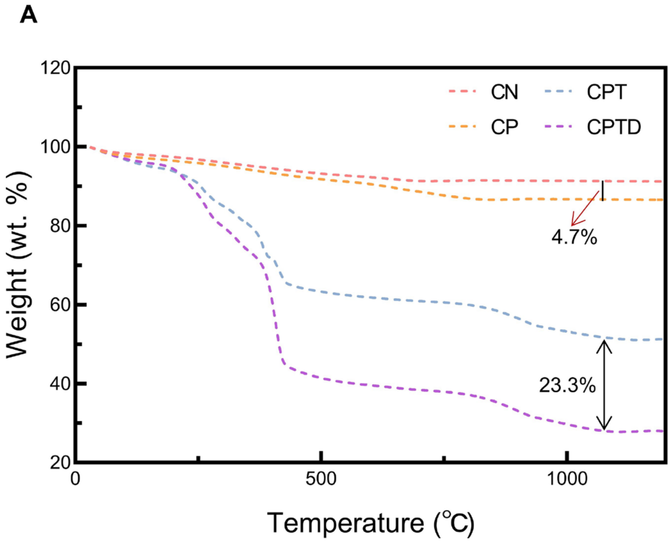

Figure S7. (**A**) TGA curves of CN, CP, CPT, and CPTD.

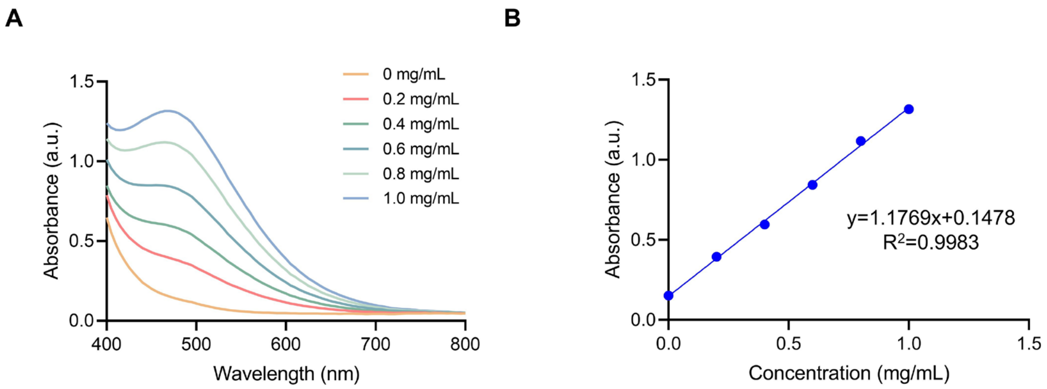

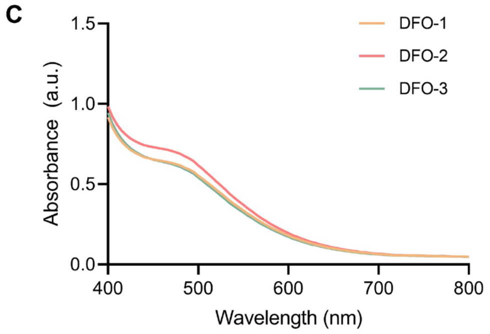

D

Encapsulation efficiency (%)	56.8 ± 3.9
Loading efficiency (%)	22.1 ± 0.1

Figure S8. (**A**) UV-vis spectra of DFO: Fe^{3+} complexes at different DFO concentrations. (**B**) Calibration curves of the adsorption peak at 420 nm at different concentrations of DFO ranging from 0 to 1.0 mg/mL. (**C**) Absorption peak at 420 nm of DFO/Fe^{3+} complex. (**D**) Encapsulation efficiency and loading efficiency of DFO in CPTD (n = 3). Data are expressed as mean ± SD.

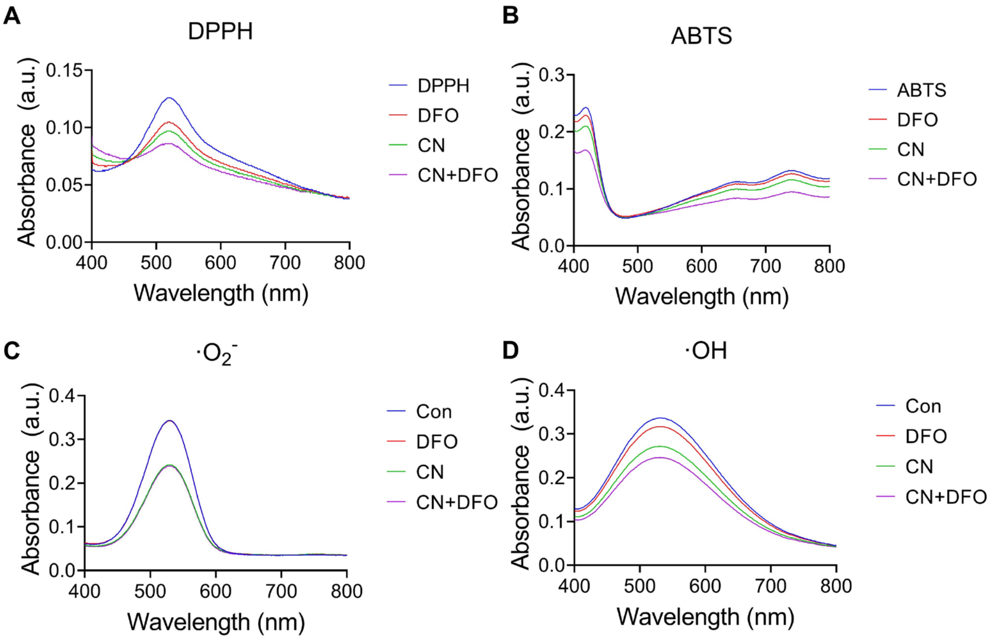

Figure S9. (**A-B**) Antioxidant capacity of CN, DFO, and CN+DFO as determined by the DPPH (**A**) and ABTS (**B**) methods. (**C**) Determination of •O_2^- scavenging capacity of CN, DFO, and CN+DFO. (**D**) Determination of •OH scavenging capacity of CN, DFO, and CN+DFO.

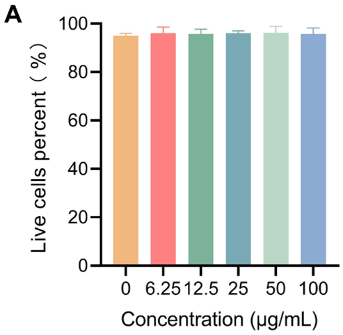

Figure S10. (**A**) Quantitative statistics of the proportion of viable cells treated with different concentrations of CPTD (n = 3). Data are presented as mean ± SD.

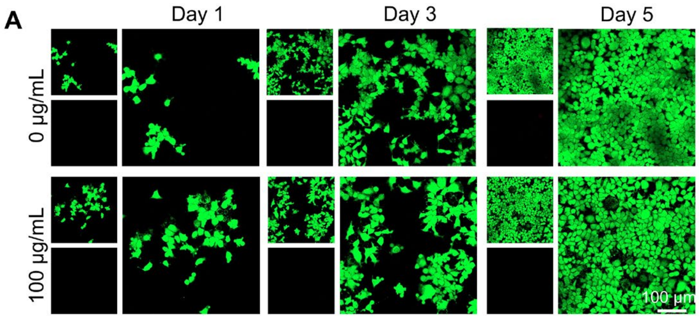

Figure S11. (A) Live/dead staining of AML12 cells at different incubation times with CPTD.

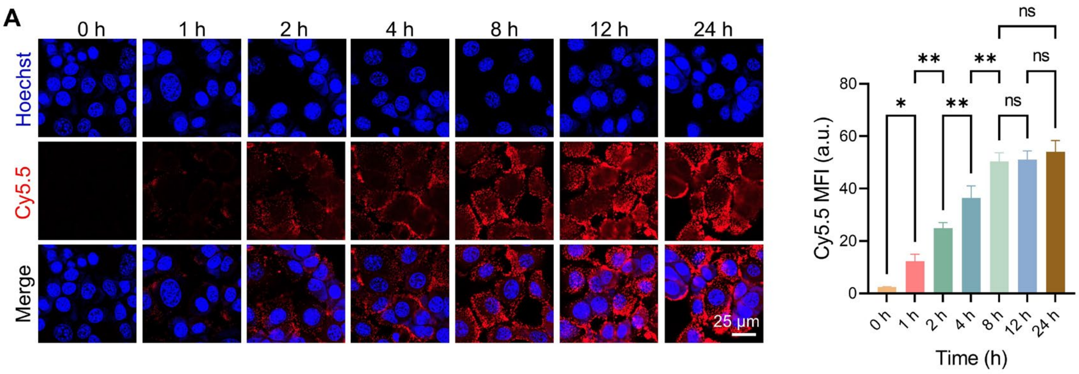

Figure S12. (A) Fluorescence imaging and quantitative analysis of Cy5.5-CPTD uptake by AML12 cells within 24 hours.

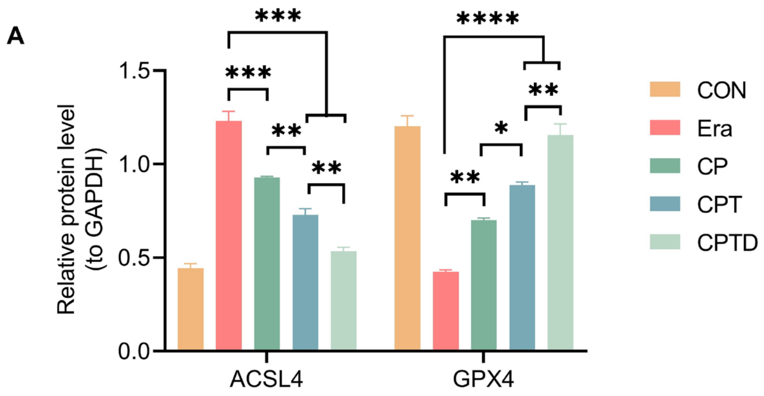

Figure S13. (**A**) Quantitative statistics of ACSL4 and GPX4 expression in AML12 cells treated with different preparations (n = 3). Data are presented as mean ± SD, n.s. not significant, *$p < 0.05$, **$p < 0.01$, ***$p < 0.001$, and ****$p < 0.0001$ (one-way ANOVA with Tukey's post hoc test).

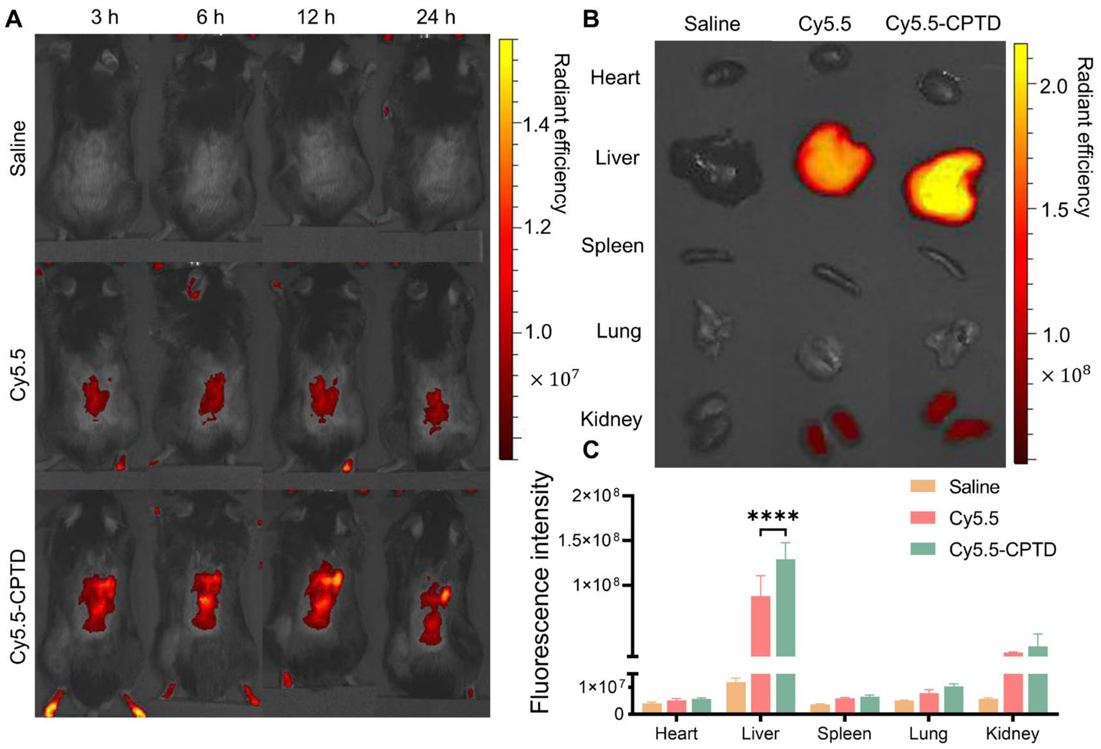

Figure S14. In vivo distribution of CPTD. (**A**) In vivo fluorescence imaging of mice at the indicated time points after intravenous injection of Cy5.5 or Cy5.5-CPTD. (**B-C**) Biodistribution and quantification of Cy5.5 and Cy5.5-CPTD in major organs (heart, liver, spleen, lungs, and kidneys) 24 h after intravenous injection (n = 3 per group). Scaled color, Min = 1.0×10^8, Max = 2.1×10^8. All images acquired with the same detection conditions, exposure time, and excitation light power. Data are presented as mean ± SD, $^{****}p < 0.0001$ (one-way ANOVA with Tukey's post hoc test).

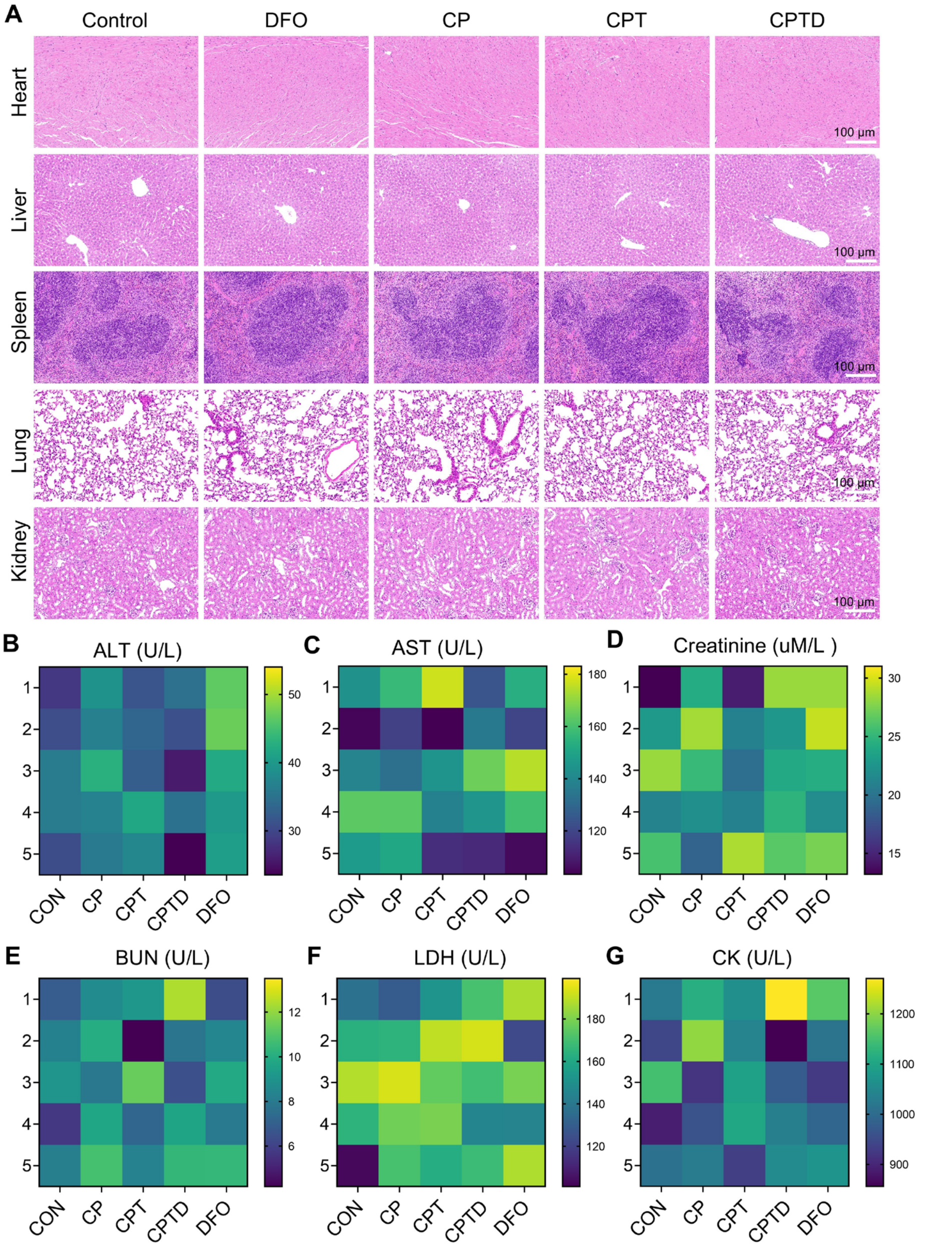

Figure S15. In vivo biocompatibility of CPTD. (**A**) Representative H&E staining images of major organs (heart, liver, spleen, lungs, and kidneys). (**B-G**) Serum ALT (**B**), AST (**C**), creatinine (**D**), BUN (**E**), LDH (**F**) and CK (**G**) levels in each treatment group (n = 5).

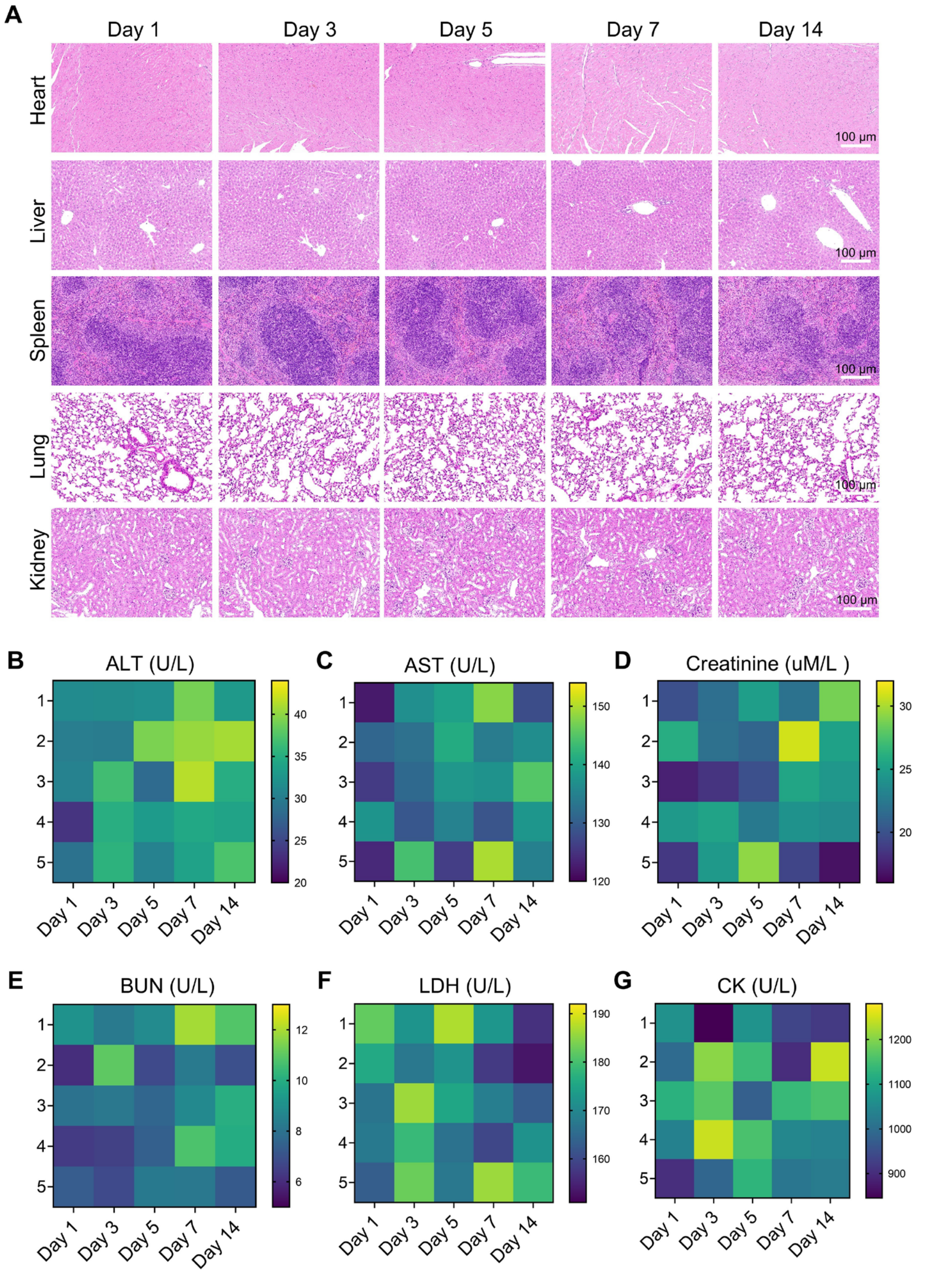

Figure S16. 14-day in vivo biocompatibility assay. (A) H&E staining of major organs including heart, liver, spleen, lungs and kidneys after received injections of CPTD. Scale bar: 100 μm. (B-G) Serum levels of ALT, AST, Cr, BUN, LDH and CK (day 14).

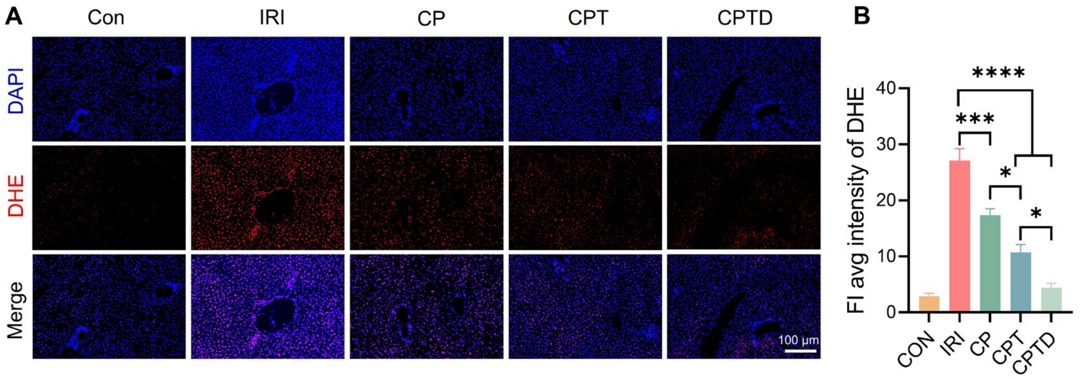

Figure S17. (**A-B**) Representative images of DHE staining of liver sections (**A**) and quantification of fluorescence intensity (**B**, n = 5). Data are presented as mean ± SD, n.s. not significant, *$p < 0.05$, **$p < 0.01$, ***$p < 0.001$, and ****$p < 0.0001$ (one-way ANOVA with Tukey's post hoc test).

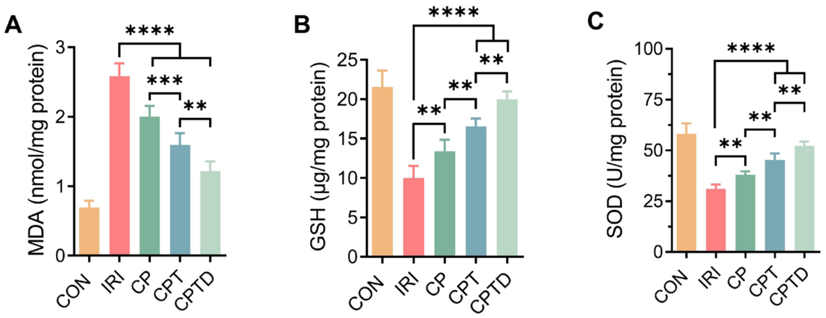

Figure S18. (**A-C**) Levels of MDA (**A**), GSH (**B**) and SOD (**C**) in liver tissue homogenates (n = 6) of IRI model. Data are presented as mean ± SD, n.s. not significant, **p* < 0.05, ***p* < 0.01, ****p* < 0.001, and *****p* < 0.0001 (one-way ANOVA with Tukey's post hoc test).

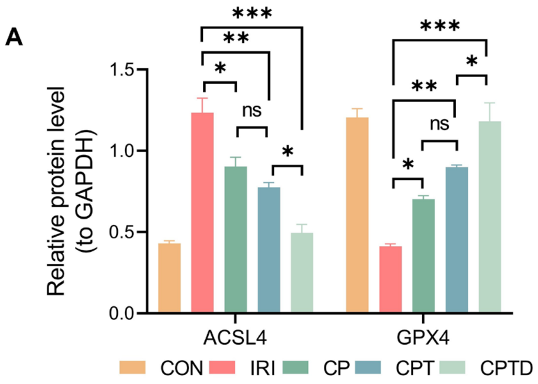

Figure S19. (**A**) Quantitative statistics of ACSL4 and GPX4 in liver tissues of mice treated with different preparations (n = 3) of IRI model. Data are presented as mean ± SD, n.s. not significant, *$p < 0.05$, **$p < 0.01$, ***$p < 0.001$, and ****$p < 0.0001$ (one-way ANOVA with Tukey's post hoc test).

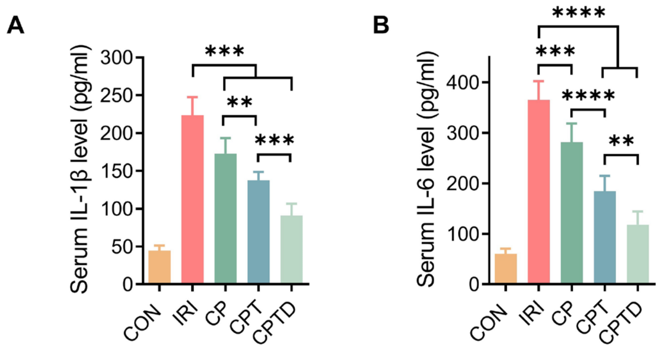

Figure S20. (**A-B**) ELISA quantification of IL-1β (**A**) and IL-6 (**B**) in serum of mice in each treatment group (n = 6) of IRI model. Data are presented as mean ± SD, n.s. not significant, *$p < 0.05$, **$p < 0.01$, ***$p < 0.001$, and ****$p < 0.0001$ (one-way ANOVA with Tukey's post hoc test).

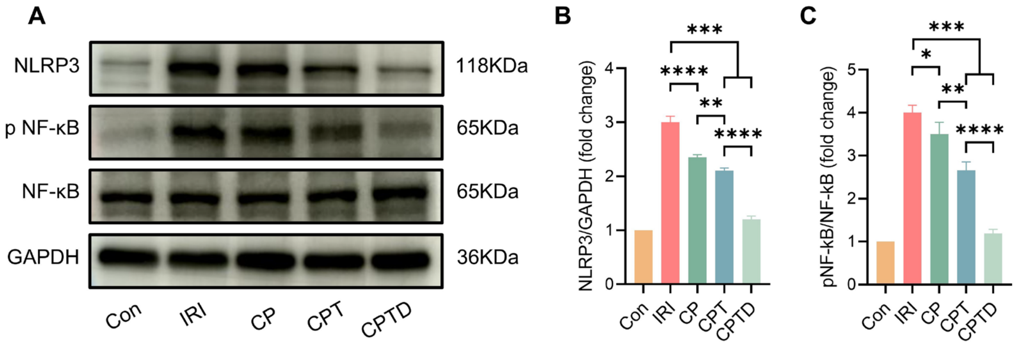

Figure S21. (A) Western blot analysis of NLRP3, p NF-κB, and NF-κB expression after I/R and treatment. (B) Quantitative statistics of NLRP3 and p NF-κB in liver tissues of mice treated with different preparations (n = 3) of IRI model. Data are presented as mean ± SD, n.s. not significant, *$p < 0.05$, **$p < 0.01$, ***$p < 0.001$, and ****$p < 0.0001$ (oneway ANOVA with Tukey's post hoc test).

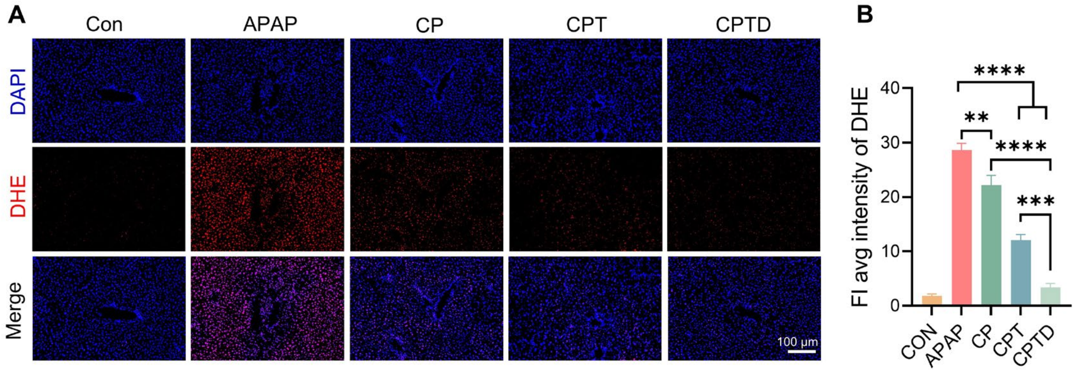

Figure S22. (**A-B**) Representative images of DHE staining of liver sections (**A**) and quantification of fluorescence intensity (**B**, n = 5). Data are presented as mean ± SD, n.s. not significant, *p < 0.05, **p < 0.01, ***p < 0.001, and ****p < 0.0001 (one-way ANOVA with Tukey's post hoc test).

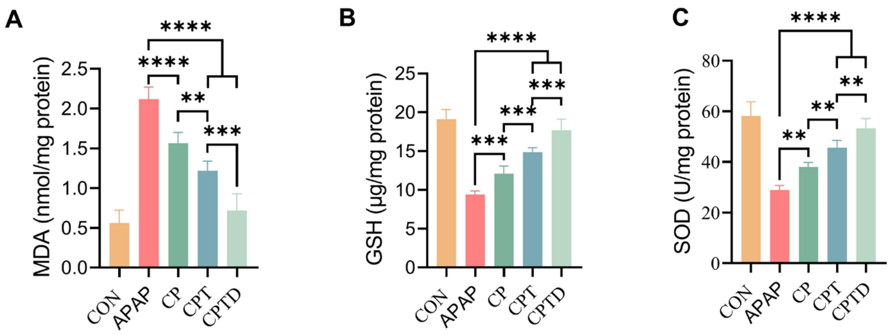

Figure S23. (**A-C**) Levels of MDA (**A**), GSH (**B**) and SOD (**C**) in liver tissue homogenates (n = 6) of APAP- induced ALI model. Data are presented as mean ± SD, n.s. not significant, *$p < 0.05$, **$p < 0.01$, ***$p < 0.001$, and ****$p < 0.0001$ (one-way ANOVA with Tukey's post hoc test).

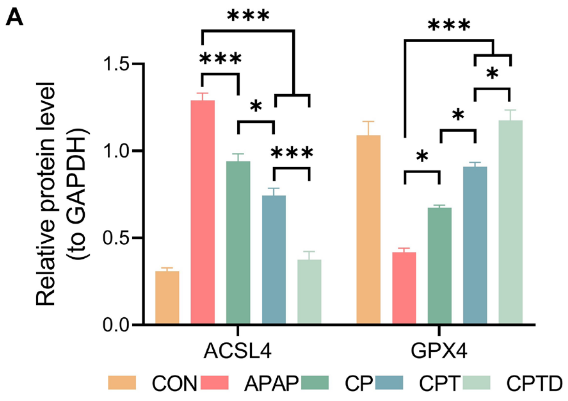

Figure S24. (**A**) Quantitative statistics of ACSL4 and GPX4 in liver tissues of mice treated with different preparations (n = 3) of APAP- induced ALI model. Data are presented as mean ± SD, n.s. not significant, *$p < 0.05$, **$p < 0.01$, ***$p < 0.001$, and ****$p < 0.0001$ (one-way ANOVA with Tukey's post hoc test).

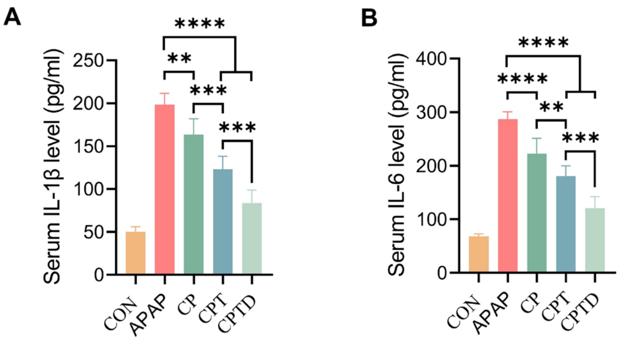

Figure S25. (**A-B**) ELISA quantification of IL-1β (**A**) and IL-6 (**B**) in serum of mice in each treatment group (n = 6) of APAP- induced ALI model. Data are presented as mean ± SD, n.s. not significant, *p < 0.05, **p < 0.01, ***p < 0.001, and ****p < 0.0001 (one-way ANOVA with Tukey's post hoc test).

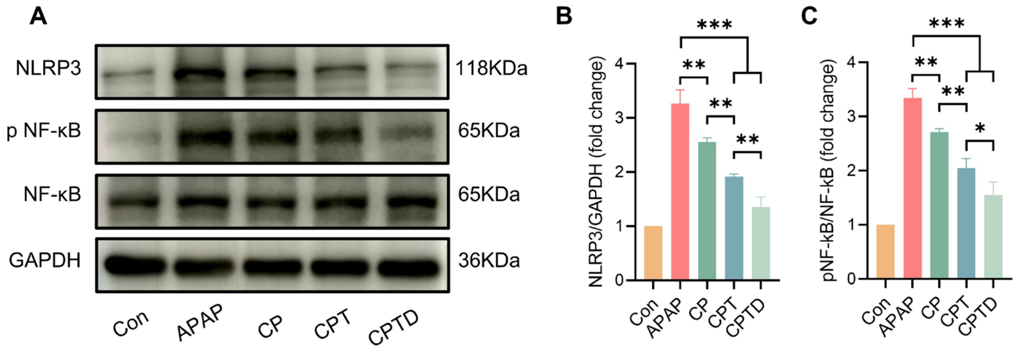

Figure S26. (A) Western blot analysis of NLRP3, p NF-κB, and NF-κB expression after APAP intoxication and treatment. (B) Quantitative statistics of NLRP3 and p NF-κB in liver tissues of mice treated with different preparations (n = 3) of APAP- induced ALI model. Data are presented as mean ± SD, n.s. not significant, *p < 0.05, **p < 0.01, ***p < 0.001, and ****p < 0.0001 (oneway ANOVA with Tukey's post hoc test).

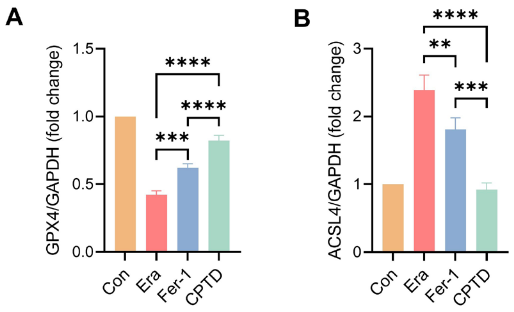

Figure S27. (**A-B**) Quantitative statistics of ACSL4 and GPX4 expression in AML12 cells treated with different preparations (n = 3). Data are presented as mean ± SD, n.s. not significant, *$p < 0.05$, **$p < 0.01$, ***$p < 0.001$, and ****$p < 0.0001$ (one-way ANOVA with Tukey's post hoc test).

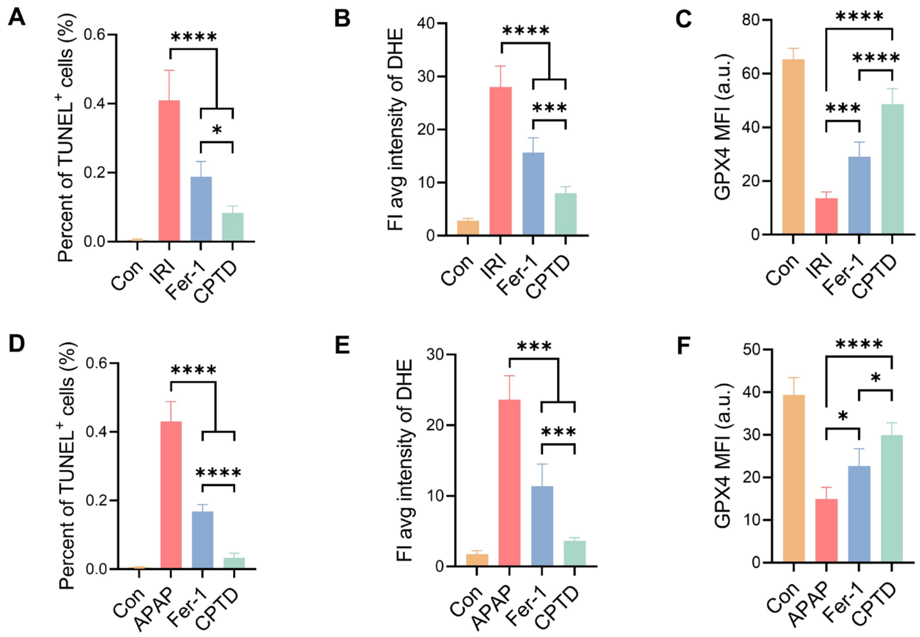

Figure S28. (**A**) Quantitative statistics of the number of TUNEL-positive cells in IRI model (n = 5). (**B**) Quantification of DHE fluorescence intensity in IRI model (n = 5). (**C**) GPX4 fluorescence intensity of mouse liver sections in IRI model (n = 5). (**D**) Quantitative statistics of the number of TUNEL-positive cells in DILI model (n = 5). (**E**) GPX4 fluorescence intensity of mouse liver sections in DILI model (n = 5). (**F**) Quantification of DHE fluorescence intensity in DILI model (n = 5). Data are presented as mean ± SD, n.s. not significant, *$p < 0.05$, **$p < 0.01$, ***$p < 0.001$, and ****$p < 0.0001$ (one-way ANOVA with Tukey's post hoc test).
